# Mapping chromatin structure at base-pair resolution unveils a unified model of *cis*-regulatory element interactions

**DOI:** 10.1016/j.cell.2025.10.013

**Published:** 2025-11-05

**Authors:** Hangpeng Li, James L.T. Dalgleish, George Lister, Maria Julia Maristany, Jan Huertas, Ana M. Dopico-Fernandez, Joseph C. Hamley, Nicholas Denny, Gianna Bloye, Weijiao Zhang, Lance Hentges, Roman Doll, Ye Wei, Michela Maresca, Emilia Dimitrova, Lior Pytowski, Edward A.J. Tunnacliffe, Mira Kassouf, Doug Higgs, Elzo de Wit, Robert J. Klose, Lothar Schermelleh, Rosana Collepardo-Guevara, Thomas A. Milne, James O.J. Davies

**Affiliations:** 1 MRC Molecular Haematology Unit, MRC Weatherall Institute of Molecular Medicine, Radcliffe Department of Medicine, University of Oxford, Oxford OX3 9DS, UK; 2 MRC WIMM Centre for Computational Biology, MRC Weatherall Institute of Molecular Medicine, University of Oxford, Oxford OX3 9DS, UK; 3 Department of Biochemistry, University of Oxford, Oxford OX1 3QU, UK; 4 Maxwell Centre, Cavendish Laboratory, Department of Physics, University of Cambridge, Cambridge CB3 0HE, UK; 5 Yusuf Hamied Department of Chemistry, University of Cambridge, Cambridge CB2 1EW, UK; 6 Department of Genetics, University of Cambridge, Cambridge CB2 3EH, UK; 7 Division of Gene Regulation, the Netherlands Cancer Institute, 1066 CX Amsterdam, the Netherlands; 8 Sir William Dunn School of Pathology, University of Oxford, Oxford OX1 3RE, UK; 9 Pixel Biology Ltd., Oxford, UK; 10 MRC Weatherall Institute of Molecular Medicine, University of Oxford, Oxford OX3 9DS, UK; 11 Chinese Academy of Medical Sciences Oxford Institute, University of Oxford, Oxford OX3 7BN, UK; 12 National Institute of Health Research Blood and Transplant Research Unit in Precision Cellular Therapeutics, University of Oxford, Oxford OX3 9DU, UK; 13 Oxford National Institute of Health Research Biomedical Research Centre, University of Oxford, Oxford OX3 9DU, UK

## Abstract

Chromatin structure is a key determinant of gene expression in eukaryotes, but it has not been possible to define the structure of *cis*-regulatory elements at the scale of the proteins that bind them. Here, we generate multidimensional chromosome conformation capture (3C) maps at base-pair resolution using Micro Capture-C ultra (MCCu). This can resolve contacts between individual transcription factor motifs within *cis*-regulatory elements. Using degron systems, we show that removal of Mediator complex components alters fine-scale promoter structure and that nucleosome depletion plays a key role in transcription factor-driven enhancer-promoter contacts. We observe that chromatin is partitioned into nanoscale domains by nucleo-some-depleted regions. This structural conformation is reproduced by chemically specific coarse-grained molecular dynamics simulations of the physicochemical properties of chromatin. Combining MCCu with molecular dynamics simulations and super-resolution microscopy allows us to propose a unified model in which the biophysical properties of chromatin orchestrate contacts between *cis*-regulatory elements.

## Graphical abstract

**Graphical abstract:**
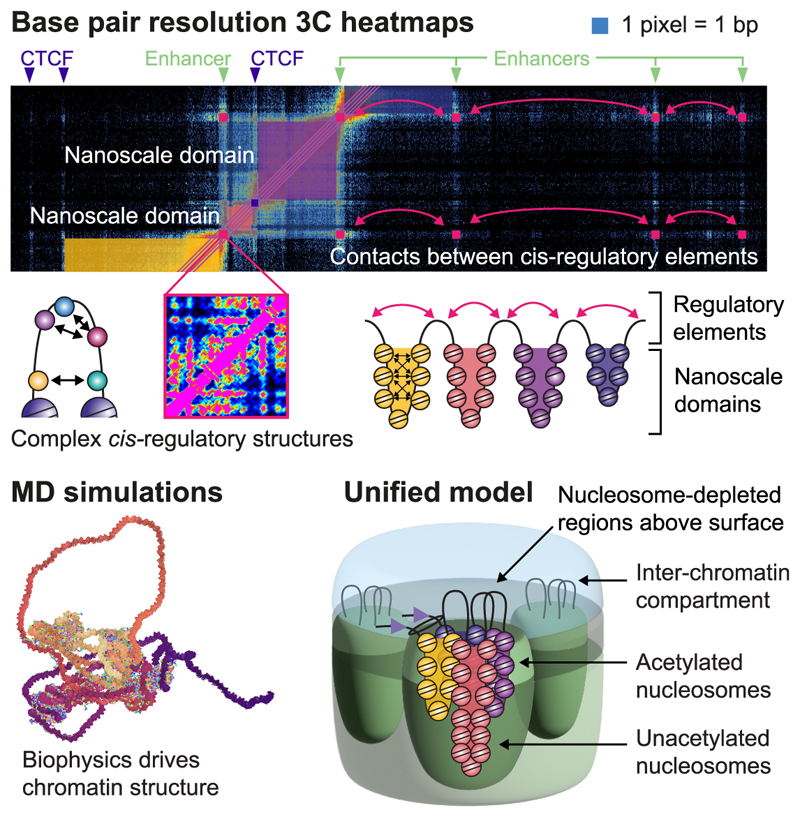

## Introduction

The evolution of complex mechanisms to allow precise spatial and temporal control of gene expression is one of the key drivers for the development of complex multicellular organisms. In higher eukaryotes many genes are regulated by multi-protein complexes bound to small (200–500 bp) *cis*-regulatory elements (enhancers, promoters, and insulators), which may be separated by 1–1,000s of kb of DNA in the linear genome.^1^

Over the past 20 years, our understanding of the relationship between genome structure and function has greatly increased, mainly driven by the ever-increasing resolution of new techniques in super-resolution imaging and chromosome conformation capture (3C). These approaches have revealed ever more detailed levels of chromatin organization from chromosomal territories to chromatin compartments, topologically associating domains (TADs), sub-TADs, and fine-scale contacts between *cis*-regulatory elements.^1–3^ Current 3C methods and imaging have shown at high resolution that when active, specific *cis*-elements often come into close physical proximity (~200–300 nm).^4–6^ However, the mechanisms by which multiple *cis*-regulatory elements come into physical proximity are not fully understood.^4^

Using previously developed Micro Capture-C (MCC),^7^ we demonstrated that long-range physical contacts occur precisely between regulatory elements at the resolution of individual transcription factors. However, MCC data only define contact frequencies from single points in the genome, whereas contact frequencies need to be determined from all positions within the region of interest to delineate detailed structures. All current (3C) approaches that generate this type of “all vs. all” data, such as Hi-C,^8^ Micro-C,^9–11^ and Region Capture Micro-C (RCMC),^12^ are unable to resolve the chromatin structures below 200 bp. Thus, it has not been possible to define DNA structure *in vivo* at the scale of the nucleosomes, transcription factors, and the protein complexes such as the Mediator complex, which control RNA polymerase II activity. This means that it has been challenging to link 3C data to the biochemical and biophysical processes controlling transcription.

The ultimate aim of further developing new approaches to analyze the chromatin structure of the genome is to understand how protein complexes form at *cis*-regulatory elements, how they interact with one another, and how these interactions impact the transcription cycle to regulate gene expression. Here, we have developed a new approach to address this issue, which provides data at extremely high resolution even down to a single base-pair pixel size. This approach has revealed fine-scale structures within nucleosome-depleted regions at individual *cis*-regulatory elements, which are likely driven by transcription factor binding. In addition, it allows nucleosome positioning to be determined and shows how long-range interactions may be perturbed by changes in the transcription factor and co-factor binding at regulatory elements. Data at this resolution have also allowed us to link 3C data with the biophysical properties of chromatin using advanced molecular dynamics simulations.

## Results

### MCCu generates data with significantly greater resolution than other available methods

**Figure 1:**
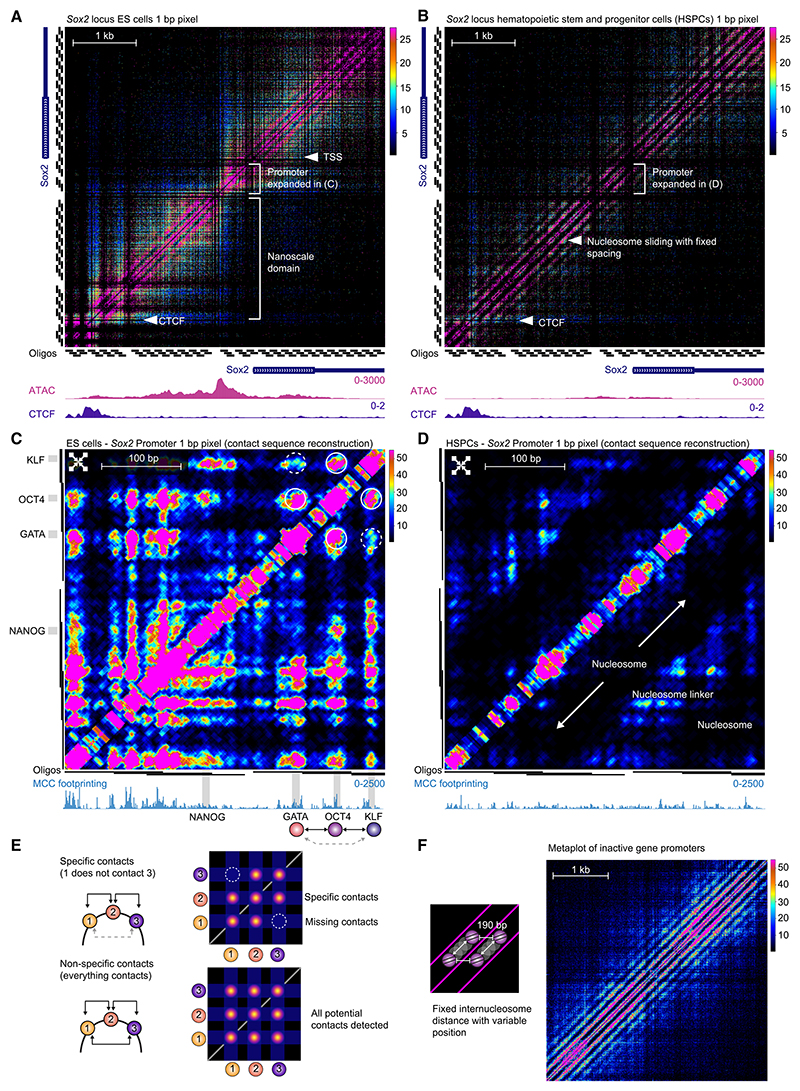
Base-pair resolution contact matrices of the *Sox2* promoter (A) Contact matrix of the *Sox2* promoter region at 1-bp resolution in embryonic stem (ES) cells in which the gene is expressed (iterative correction and eigenvector decomposition [ICE] normalized matrices of read junction density; 6 replicates). Capture oligonucleotide positions, assay for transposase-accessible chromatin sequencing (ATAC-seq), and chromatin immunoprecipitation sequencing (ChIP-seq) for CTCF are shown below the contact maps (read density counts per million mapped reads [CPM]). In the active state, the promoter is split into two nanoscale domains by the central nucleosome-depleted region. (B) Contact matrix of the *Sox2* promoter region at 1-bp resolution in HSPCs, in which it is inactive. The diagonal lines of high signal at the inactive promoter represent ligation junctions between adjacent local nucleosome linkers, which have fixed internucleosomal spacing but variable position relative to DNA sequence. (C) Contact sequence reconstruction maps (see Figure S2A) of the nucleosome-depleted region at the *Sox2* promoter in ES cells (*cis*-normalized directional vector densisty). Transcription factor motifs annotated with JASPAR database^13^ and ChIP-seq.^14–19^ White circles highlight contact points between transcription factor binding sites, and the dashed circle highlights an absent contact point. (D) Contact sequence reconstruction maps at the same site as (C) but in HSPCs in which the horizontal line of junctions delineates nucleosome linker positions in keeping with the repressed state of *Sox2* in HSPCs. (E) Model of specific vs. non-specific contacts between elements. Specific contacts result in distinct interaction points and regions of absent signal. Non-specific contacts occur when all elements interact with one another. (F) Metaplot of ICE normalized read junction density for inactive gene promoters showing pattern generated by nucleosomes with a variable position but fixed internucleosome distance. See also Figure S1.

Here, we present MCC ultra (MCCu), which allows multidimensional contact maps to be generated with a single base-pair pixel size (Figure 1A). Generating maps at this resolution is challenging because a 10-fold increase in resolution requires a 100-fold increase in data (increasing resolution by a factor of *n* requires an *n*^2^ increase in read depth^8^). In addition, production of heatmaps is challenging, compared with individual viewpoint profiles, because every row in the matrix is equivalent to a profile from an individual viewpoint (a 5-kb heatmap with a 1-bp pixel size contains 5,000 individual profiles).

In conventional 3C approaches, including the original version of MCC, very short-range contact data are excluded from analysis because the short-range signals are huge, compared with the contacts with distal regulatory elements. However, we realized that the vast number of short-range ligation junctions in MCC libraries contains a large amount of information about the fine-scale chromatin structure that can be used to generate extremely high-resolution Hi-C like maps of regions of interest. This is not possible with other 3C methods because they do not provide short-range base-pair resolution ligation junction data. This was accomplished by single fixation with 2% formal-dehyde and the use of digitonin in place of stronger detergents, combined with sonicating the 3C library to 200 bp and subsequently sequencing with 300 bp reads to allow for full reconstruction of the sequence. In contrast to other micrococcal nuclease (MNase)-based 3C assays, the capture oligonucleotide design and bioinformatic pipeline are also optimized for precise identification of fine-scale ligation junctions (Figure S1A). By comparison, Micro-C^10^ and RCMC^12^ have an inherent limit in their resolution below 200 bp, because following proximity ligation, dinucleosomal (300–400 bp) DNA fragments are extracted, and these are sequenced with 50-bp paired-end sequencing, which means that it is not possible to determine the exact position of ligation junctions (Figure S1A).

Switching from individual viewpoints to tiling approaches requires the number of capture oligonucleotides to be increased (around ~80 oligos per viewpoint rather than one). In order to generate data of sufficient depth to achieve this resolution (equivalent to trillions of read pairs of genome wide data), we redeveloped the oligonucleotide capture protocol (STAR Methods). Analysis of the short-range data required development of a new analysis pipeline to generate heatmaps at basepair resolution, which includes functionality to generate high-resolution heatmaps from short-range contacts as well as those that extended outside of the captured region across the genome. This makes the approach a hybrid between 4C and Hi-C, with advantages of both, because it allows contacts of all the interacting partners to be characterized in detail.

To visualize the improvements made by MCCu, we plotted contact maps comparing MCCu at 6-kb regions around the *Sox2* and *Nanog* promoters in mouse embryonic stem (ES) cells, which have been extensively characterized by other methods including Micro-C and RCMC^12^ (Figures S1B–S1D). With Micro-C or RCMC it is difficult to discern contact domains below 1 kb in size even if it is plotted with a 50-bp pixel size, and below this resolution (10-bp pixel size), it is challenging to extract meaningful information (Figures S1C and S1D). In contrast, MCCu enables data to be generated with a single bp pixel size. Having established the increased resolution of our method, we used MCCu to analyze 15 gene promoters in three different murine cell types (ES cells, hematopoietic stem and progenitor cells [HSPCs], and erythroid cells).

### Base-pair resolution contact maps reveal cell-type-specific interactions between transcription factor binding sites at promoters

MCCu revealed fine-scale substructures within promoters and allowed for the positioning of nucleosomes to be delineated. For example, at the *Sox2* promoter in ES cells, we visualized highly specific contacts between adjacent nucleosome-depleted regions and CTCF sites (Figure 1A). In contrast, data from hematopoietic stem cells, in which *Sox2* is not expressed, show a signature consistent with inert chromatin (Figure 1B).

On a smaller scale (<200 bp), within nucleosome-depleted regions, MCCu delineated specific contacts between individual transcription factor motifs. MNase cuts around transcription factor binding sites, and so the raw data define fine-scale points of interaction by increased ligation junction counts at the corners surrounding the contact point, which makes it challenging to identify the sequences underlying contacts (Figure S2A). However, we exploited the fact that DNA binding proteins protect the sequence adjacent to the MNase cut site to identify whether the protein leading to the ligation junction was upstream or downstream of the ligation junction. To visualize contacts between transcription factor motifs, we developed a contact sequence reconstruction approach that uses the directionality of the reads from ligation junction pairs to reconstruct the position of sequences associated with high levels of ligation junctions onto the contact matrix (Figures S2A–S2G).

Contact sequence reconstruction revealed that within nucleosome-depleted regions, high levels of ligation junctions occur between specific sequences (Figures 1C–1E). For example, at the *Sox2* promoter, we identified a high signal between a POU5F1 (OCT4) motif and a Krüppel-like factor (KLF) family motif upstream and a GATA motif downstream. However, few ligation junctions are seen between the KLF and GATA motifs (Figure 1C). These regions of high signal are absent in HSPCs, in which the gene is not expressed (Figures 1B and 1D). Specific contacts between transcription factor binding sites have not previously been visualized at this resolution, and these data show that the DNA in nucleosome-depleted regions forms complex structures (Figure 1E).

### MCCu reveals nucleosome positioning and nanoscale domains at promoters

At active promoters, we found that nucleosome-depleted regions partition chromatin into nanoscale domains, which have a similar appearance to conventional TADs but are approximately 100- to 1,000-fold smaller. Promoters with a single nucleosome-depleted region partitioned into two domains (Figure S2H), whereas promoters, such as *Klf4* and *Myc*, which contain multiple nucleosome-depleted regions, partitioned into multiple domains (Figures 1A, S2I, and S2J).

Further analysis revealed two major patterns of nucleosome positioning. At most regions, the positioning of individual nucleosomes was not fixed, but there were strong ligation junction signals with a periodicity of ~180–190 bp corresponding to adjacent nucleosome linkers (Figures 1B, S2K, and S2L). This signal was prominent at untranscribed regions and may provide a means of actively defining inactive regions of the genome (Figure 1F). This is likely due to maintenance of internucleosomal distances in the context of non-fixed positioning of individual nucleosomes. At other sites, we were able to define fixed nucleosome positioning (Figure S2K), which often occurred adjacent to CTCF binding sites and nucleosome-depleted regions.

We went on to analyze the effects of histone acetylation on nucleosome spacing by plotting the distance between ligation junctions for regions with H3K27ac detected by chromatin immunoprecipitation sequencing (ChIP-seq). This showed that histone acetylation is associated with reduced periodicity of inter-ligation junction distances, which indicates greater variability in nucleosome spacer lengths, in keeping with *in vitro* studies showing that acetylation of histone tails results in reduced affinity for DNA (Figures S2K and S2L).^20^

### Visualization of physical proximity between transcription factor binding sites within enhancers

**Figure 2:**
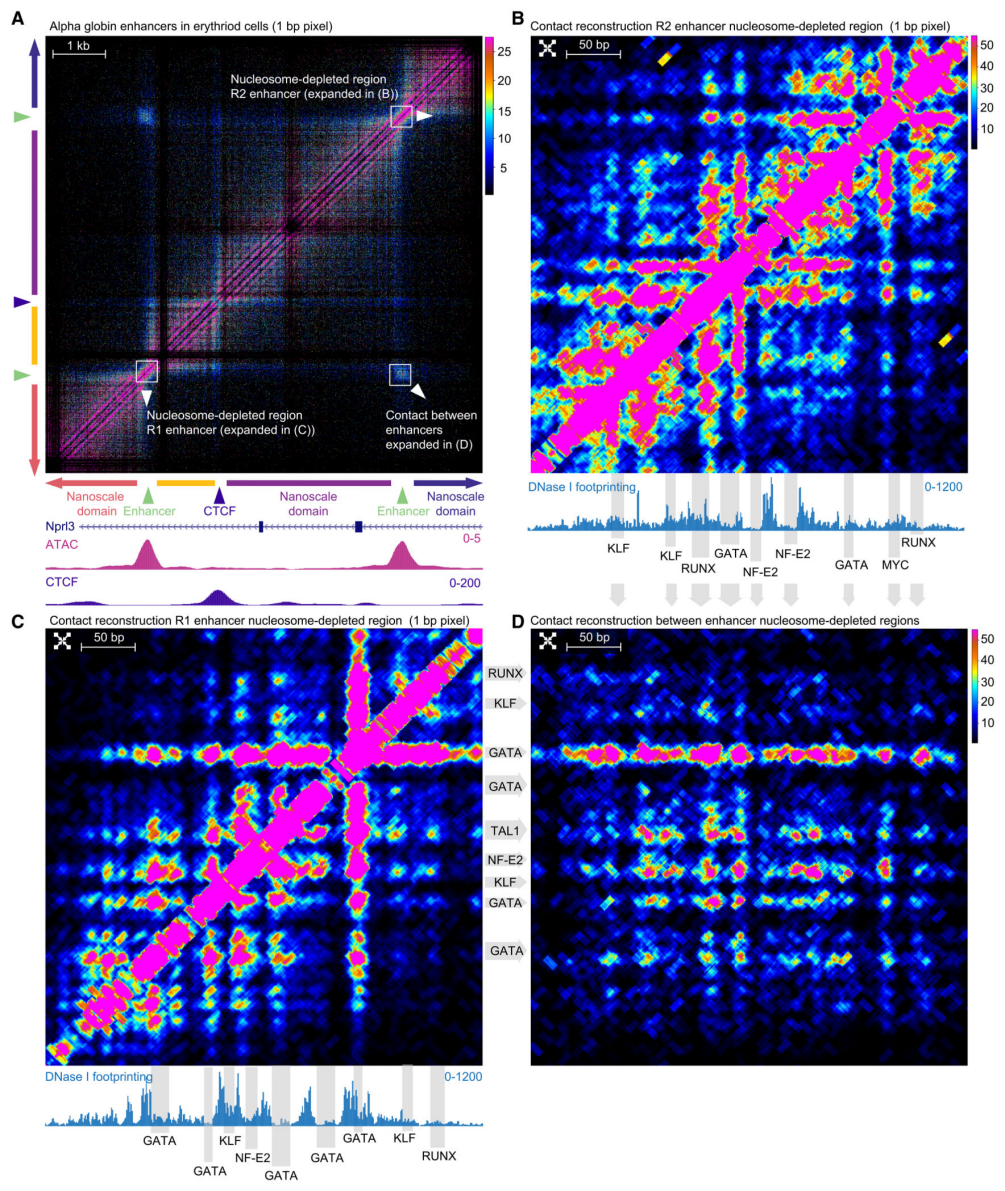
Base-pair resolution maps of *α-globin* enhancers (A) Contact matrix of the main enhancers at the alpha globin locus region at 1-bp resolution in murine Ter119+ erythroid cells (ICE normalized matrices of read junction density; 6 replicates). (B and C) Showing the enhancer regions in detail, using contact sequence reconstruction to identify signal between different transcription factor binding motifs. DNase I hypersensitivity footprinting, JASPAR,^13^ ChIP-seq, and conservation analysis^7,21,22^ are used to identify transcription factor motifs (*cis*-normalized directional vector density). (D) Contact sequence reconstruction of interactions between the two enhancers highlighted in (B) and (C) (*cis*-normalized directional vector density). See also Figure S2.

It has been shown that super-enhancers play a pivotal role in gene regulation and cellular differentiation, but their intricate structures remain unclear. We, therefore, interrogated the structures of five super-enhancers at well-characterized loci (alpha globin [*Hba-a1&2*] in erythroid cells and *Myc, Sox2, Nanog*, and *Tet2* in ES cells). Similar to promoters, enhancers formed complex structures characterized by specific contacts between nucleosome-depleted regions containing transcription factor binding sites. In many cases, CTCF played a role in modifying small-scale structures. We also observed partitioning of the chromatin into nanoscale domains between the nucleosome-depleted regions containing transcription factor binding sites (Figure 2A).

A notable example is observed in the alpha globin superenhancer region, where the two most critical elements make highly punctate contacts (Figure 2A).^21,23^ Specifically, within the nucleosome-depleted regions at enhancers, we delineated distinct contacts between transcription factor binding sites by using contact sequence reconstruction (Figures 2B, 2C, and S2A–S2G). We identified strong contacts from GATA::TAL1 binding sites, which are known to recruit LDB1 and to form a protein complex that plays an important role in erythropoiesis (Figure 2D).^24,25^

### Enhancer-promoter contacts occur precisely between nucleosome-depleted regions

The mechanisms responsible for enhancer-promoter interactions are not fully understood.^4^ Previous studies have proposed different models including specific interactions mediated by DNA binding proteins, loop extrusion,^26,27^ and condensates driven by liquid-liquid phase separation.^28,29^ We, therefore, sought to visualize enhancer-promoter contacts at high resolution. To achieve this, we further increased the depth of data (4-fold), by restricting the region interrogated to a single promoter or enhancer, which allowed high concentrations of individually synthesized biotinylated oligonucleotides to be used.

**Figure 3:**
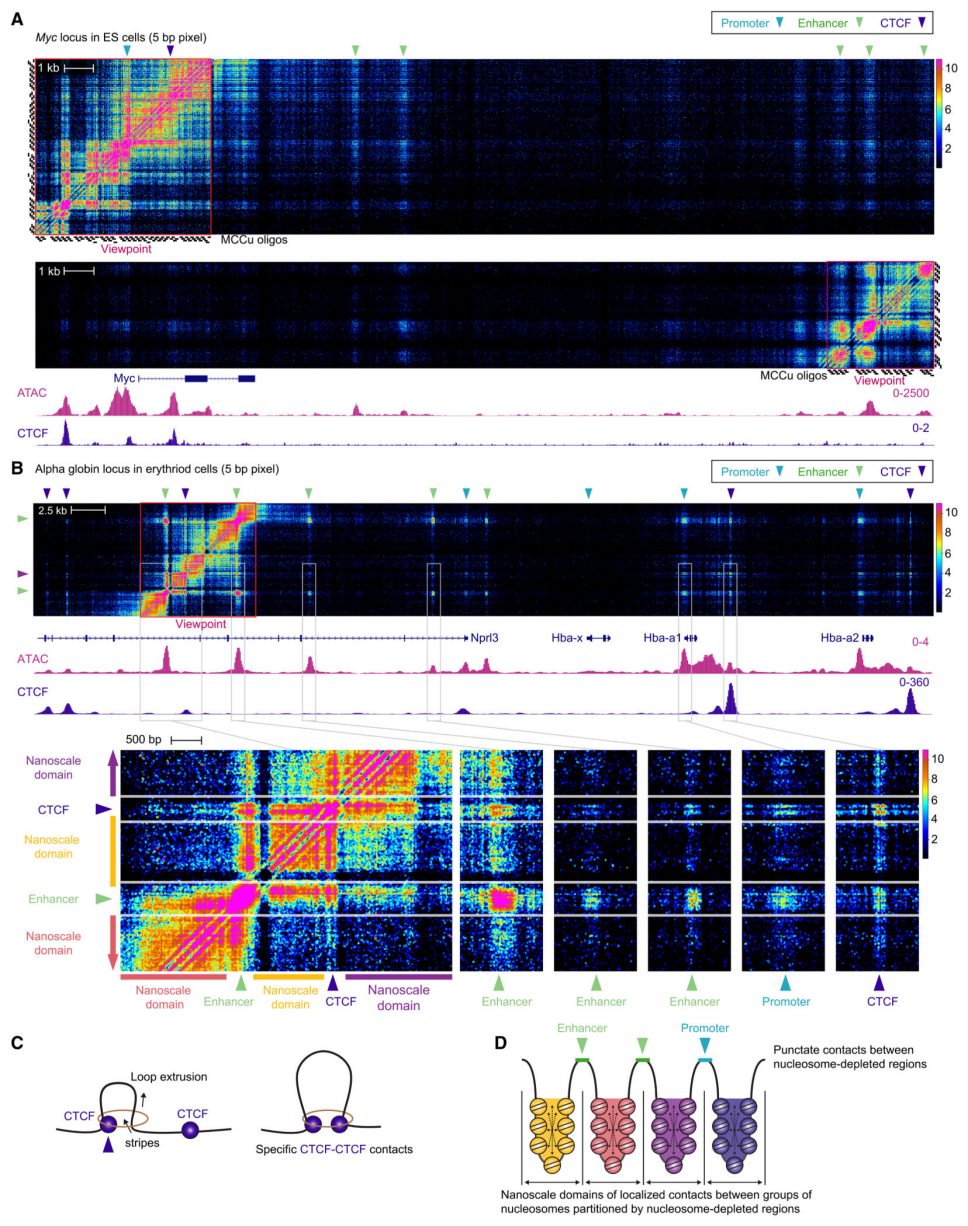
High-resolution maps of the enhancer-promoter contact (5-bp pixel size) (A) Heatmaps of enhancer-promoter contacts at the *Myc* locus. The upper heatmap illustrates the promoter contacts (viewpoint highlighted with red box), while the lower heatmap presents the reciprocal interactions from the enhancers (5-bp pixel size; junction density *cis*-normalized for oligo capture efficiency; 6 replicates). Capture oligonucleotide positions, ATAC-seq, and ChIP-seq for CTCF are shown below the heatmaps. (B) Heatmap of the contacts from the main enhancers at the alpha globin locus in murine Ter119+ erythroid cells (5-bp pixel size; junction density *cis*-normalized for oligo capture efficiency; 6 replicates). Shown below is magnification of the parts of the heatmap. The CTCF binding site makes a specific contact with intergenic CTCF sites and stripes can be seen, likely due to loop extrusion (C). (C) Schematic of loop extrusion. (D) Schematic of the model in which the nucleosome-depleted regions contact one another, and the nucleosome associated chromatin forms nanoscale domains of interactions, which are bound by nucleosome-depleted regions. See also Figure S3.

With this approach, combined with the new bioinformatics pipeline, which allows genome wide heatmaps to be generated from regions of interest, we were able to generate maps of enhancer-promoter contacts at 5-bp resolution (Figures 3A and 3B). Analysis of both the promoter and enhancers at *Myc* in ES cells and alpha globin (*Hba-a1&2*) in erythroid cells showed that the highest density of interactions is within the promoter or enhancer regions themselves. This analysis revealed that the nucleosome-depleted regions at enhancers make highly localized contacts with the nucleosome-depleted regions at promoters. However, in contrast to small-scale contacts within nucleosome-depleted regions, we did not observe strongly specific contacts between any particular regulatory element and/or promoter (Figures 1D, 3A, and 3B). By contrast, we observed that CTCF binding sites made highly specific contacts with other CTCF binding sites rather than enhancers, in keeping with loop extrusion models (Figures 3B and 3C).^26,27,30^

At many genes, including *Myc* and *Hba-a1&2* (Figures 3A and 3B), we observed that nucleosome-depleted regions within super-enhancers contact one another more strongly than they contact gene promoters. This may be because the distances between the nucleosome-depleted regions within super-enhancers are usually smaller than the distance between the enhancer and the promoter, but it is likely that the enhancers coalesce independent of the promoter.

The extended heatmaps of many genes show that the interaction profile varies significantly depending on whether the viewpoint was directly over the nucleosome-depleted region or not (Figures 3A and 3B). Highly punctate contacts occur between nucleosome-depleted regions themselves. By contrast, the chromatin surrounding nucleosome-depleted regions was seen to make contacts that are largely constrained within a local domain containing the nearby nucleosomes and extending up to the next nucleosome-depleted region (Figures 3A, 3B, and 3D). We confirmed this using conventional MCC with 120-bp viewpoints from the promoter of genes and regions 1 kb up and downstream of the promoter. These data show that the promoters make highly punctate contacts whereas the off-set viewpoints made generalized contacts, which are largely confined within the domain up to the next nucleosome-depleted region (Figures S3A–S3D). We find that nucleosome-depleted regions make highly localized contacts with one another, and they simultaneously cause partitioning of the intervening chromatin into nanoscale domains, potentially through disruption of the biophysical properties of the chromatin (Figure 3D).

### CTCF binding sites are associated with regions of fixed nucleosome positioning and localized partitioning

CTCF binding sites are commonly located within enhancers and promoters. Although there is evidence that CTCF can play a role in gene activation,^31^ its role at regulatory elements is not completely characterized.

Using MCCu, we found that CTCF sites strongly influence the nanoscale topology of regulatory elements, and they often lead to partitioning into nanoscale domains (Figures S3E and S3F). We identified that the nucleosomes surrounding CTCF binding sites generally hold fixed positions. This was particularly prominent at sites where the CTCF site was closely positioned to another nucleosome-free region, where the intervening nucleosome pattern suggested fixed positioning of multiple nucleosomes relative to the DNA sequence (Figure S3E). These arrangements were accompanied by robust insulation between the regions upstream and downstream of the CTCF binding site (Figures S3E and S3F). We also found that CTCF binding sites were associated with highly localized stripes in the chromatin (Figures 3B and 3C). These data are in agreement with observations from other groups and are likely due to cohesinmediated loop extrusion.^26,27,32^

### Depletion of Mediator components leaves large-scale contacts intact but causes disruption of nanoscale topology within nucleosome-depleted regions

The Mediator complex plays a fundamental role in transcriptional regulation. Since structural studies have shown that the tail module of the complex interacts with transcription factors and the middle and head modules contact the pre-initiation complex (PIC), it has been postulated that it might mediate physical contacts between enhancers and promoters.^33–35^ However, previous 3C studies have reported inconsistent results,^36–38^ and it is not clear to what extent the physical contacts between enhancers and promoters are dependent on the Mediator complex at different scales.

To study this, we employed the FKBP12^F36V^ degradation tag (dTAG) system^39^ in ES cells with homozygous tagging of endogenous Mediator components (Figure S4A). The heterobifunctional dTAG molecule (dTAG-13) was applied to engage FKBP12^F36V^ with cereblon (CRBN) E3 ubiquitin ligase, which specifically targets the tagged protein and leads to rapid degradation (Figure S4B).

**Figure 4:**
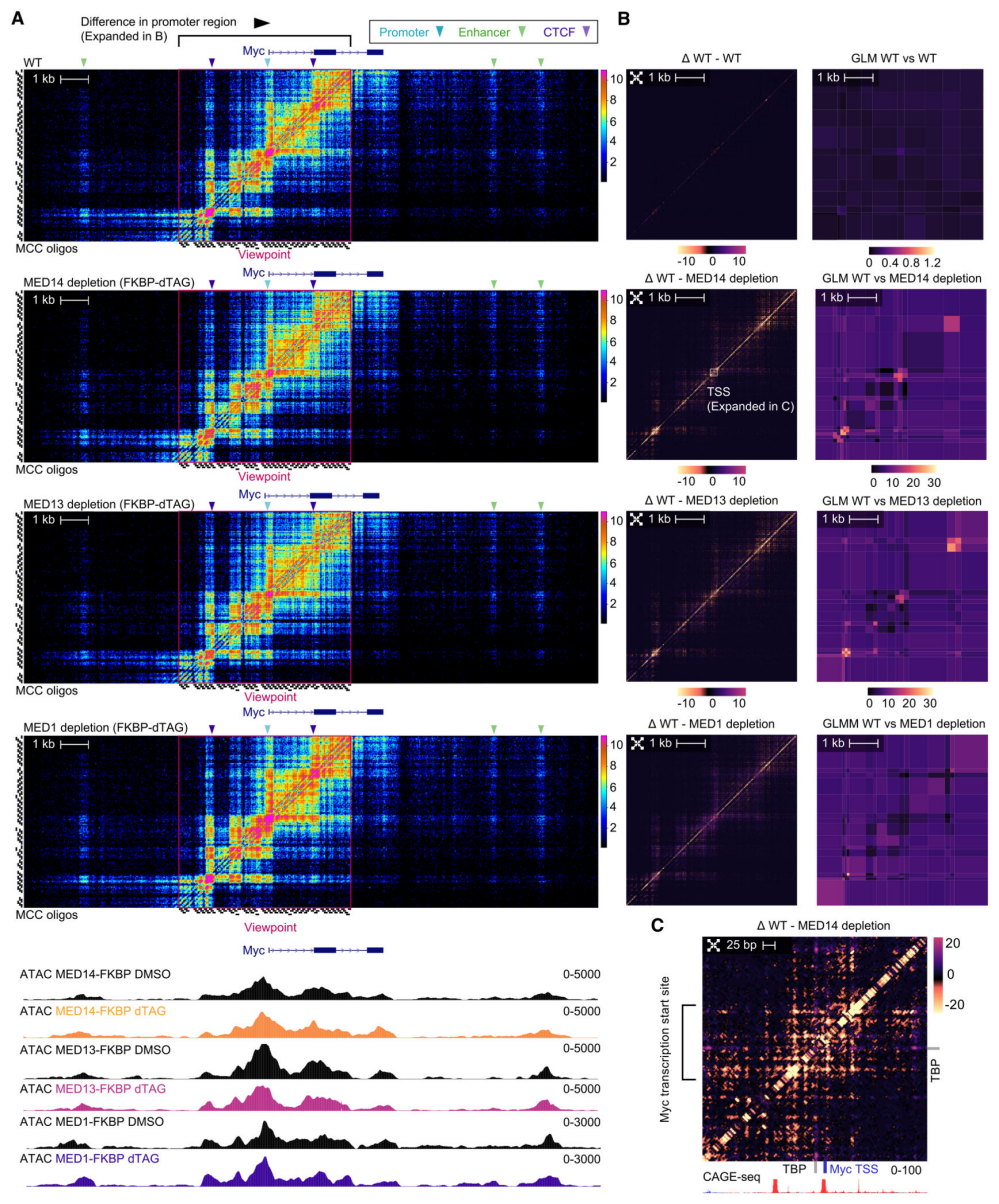
Effects of acute depletion of Mediator using the FKBP12^F36V^ dTAG degron system (A) MCCu at the *Myc* promoter in ES cells; wild type (WT), MED14-FKBP, MED13-FKBP, and MED1-FKBP cells 2 h post treatment with dTAG-13 (5-bp pixel size; junction density *cis*-normalized for oligo capture efficiency; 6 replicates). ATAC-seq data (read density [CPM]) from DMSO and dTAG-13 treatments for MED14-FKBP, MED13-FKBP, and MED1-FKBP are shown below the heatmaps. (B) Normalized differential contact matrix between WT and dTAG-13-treated cells for the viewpoint region highlighted in red in (A) (left). Analysis of the data with the statistical package basepairC (right), which includes normalization of data and correction for MNase cut site biases (normalized count difference). (C) Differential contact matrix with contact sequence reconstruction within the nucleosome-depleted region at the *Myc* promoter comparing WT with MED14 depletion (*cis*-normalized directional vector density). TBP (JASPAR database) and TSSs (FANTOM5^40^) are annotated. See also Figure S4.

Initially, we focused on MED14, which is a scaffold protein required for Mediator complex assembly. Using immunoblotting and chromatin immunoprecipitation, we confirmed that the target-selective, ligand-dependent proteolysis resulted in near-complete degradation of both whole-cell- and chromatin-associated MED14 after 2-h treatment with dTAG-13, and this did not increase with longer incubation times (Figures S4C–S4E). MED14 depletion resulted in transcriptional loss at target genes but only caused small changes in the assay for transposase-accessible chromatin sequencing (ATAC-seq) signal at regulatory elements (Figures 4A and S4F–S4H). We developed a specific statistical package (basepairC), which includes correction of MNase cut site biases, for robust comparison of datasets at this resolution.

With the increased resolution of MCCu, we observed that MED14 depletion results in significant changes between transcription factor binding sites within the nucleosome-depleted regions at promoters (Figures 4A and 4B). Specifically, the observed decreases were associated with TATA-binding protein (TBP) binding motifs located near the transcription start site (TSS) (Figures 4C and S4I), suggesting disruption of the crucial interactions needed for formation or stabilization of the transcription PIC. By comparison, we only observed subtle alterations in long-range enhancer-promoter interactions (Figures S4G and S4J).

We then went on to study the effect of acute depletion of the Mediator kinase module subunit MED13, which is essential for transcription but does not play as central a role in Mediator complex assembly as MED14. Acute degradation of MED13 with dTAG-13 resulted in complete protein depletion (Figure S4C) yet caused a less pronounced reduction in transcription, compared with the effects observed following MED14 removal (Figure S4H). Depletion of MED13 also had a significant effect on the nanoscale contacts around the TSS, similar to MED14 depletion. MED13 depletion also modestly affected large-scale chromatin topology (Figures S4G and S4J).

We also investigated the effects of acute depletion of MED1, which forms part of the middle module of the Mediator complex. In contrast to MED14 and MED13, we found that acute depletion of MED1 did not result in changes in either the small-scale interactions within the nucleosome-depleted regions at promoters or long-range contacts (Figures 4A, 4B, S4C, and S4J).

Overall, in keeping with previous lower-resolution studies,^36–38^ we find that Mediator degradation had minor effects on large-scale chromatin architecture, with most enhancer-promoter contacts remaining intact. However, depletion of MED14 and MED13 resulted in changes in chromatin structure within the nucleosome-depleted regions at gene promoters. This suggests that Mediator operates at the level of transcription complex stabilization at the promoter, rather than acting as an architectural link between promoters and enhancers to control gene expression.

### Depletion of transcription factors results in marked loss of enhancer-promoter contacts when nucleosome-depleted regions are lost

Our data show that enhancer-promoter interactions occur highly specifically between nucleosome-depleted regions. We wanted to investigate how acute depletion of two different transcription factors disrupts enhancer-promoter interactions. We again employed the FKBP12^F36V^ dTAG system (Figures S4A and S4B) to induce rapid protein depletion of key transcription factors SOX2 and NANOG in ES cells. In line with previous work,^41,42^ we found that SOX2 and NANOG are efficiently removed 2 h after addition of dTAG-13, and we confirmed reduction of transcription using transient transcriptome sequencing (TT-seq) at SOX2-dependent genes (Figure S5).

**Figure 5:**
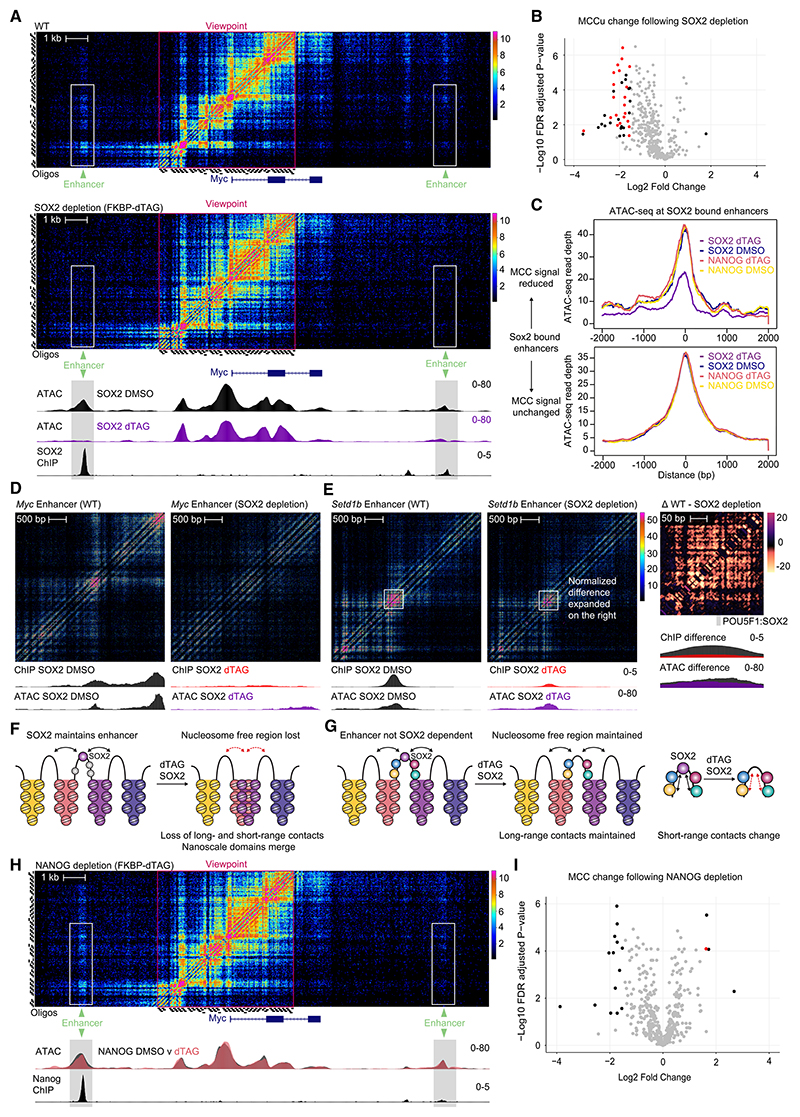
Effects of acute depletion of transcription factors using the FKBP12^F36V^ dTAG degron system (A) MCCu contact matrices of the *Myc* locus in WT ES cells; ES SOX2-FKBP 2 h post addition of dTAG-13 (5-bp pixel size; junction density *cis*-normalized for oligo capture efficiency; 6 replicates). ATAC-seq and SOX2 ChIP-seq for DMSO control and dTAG-13 treatment (read density [CPM]). (B) Comparison of MCCu junction frequencies in WT and SOX2-depleted ES cells. Interactions were called in WT ES cells using Lotron MCC. Significantly skewed peaks with SOX2 binding by ChIP-seq are shown in red (*p* value < 0.05 [adjusted for multiple testing]; absolute log_2_ fold change > 1.5). (C) Metaplots of ATAC-seq at SOX2-bound enhancers with significantly reduced MCC promoter contacts following SOX2 depletion with SOX2-bound enhancers at which there is no change in contacts. ATAC-seq for SOX2-FKBP and NANOG-FKBP cells 2 h post treatment with DMSO or dTAG-13. (D and E) Changes in enhancer topology upon depletion of SOX2 (junction density *cis*-normalized for oligo capture efficiency). Some enhancers were highly dependent on SOX2 binding, and the nucleosome-depleted region disappears on SOX2 depletion. (D) At other enhancers, SOX2 depletion did not change nucleosome occupancy, and subtle changes in local topology and long-range contacts occur. However, at these sites, MCCu revealed pronounced changes in the small-scale structure within the nucleosome-depleted region (E). (F and G) Model of variable effects of SOX2 depletion for (D) and (E), respectively. (H) MCCu contact matrix in NANOG-FKBP ES cells 2 h post addition of dTAG-13 (5-bp pixel size; junction density *cis*-normalized for oligo capture efficiency; 6 replicates). ATAC-seq from DMSO- and dTAG-13-treated cells and ChIP-seq for NANOG are shown below the heatmaps (read density [CPM]). (I) Volcano plot of effects of NANOG depletion on MCC signal. See also Figure S5.

We used MCCu to examine the effects of SOX2 depletion on enhancer-promoter interactions at 15 well-characterized promoters and 20 enhancers at which SOX2 ChIP-seq markedly reduced on dTAG treatment. MCCu detected that SOX2 degradation directly reduces interactions originating from SOX2 motifs (Figures S5G and S5H). SOX2 degradation led to a reduction in enhancer-promoter at SOX2 bound enhancers (Figure 5B). At the *Myc* locus, SOX2 depletion resulted in the collapse of nucleosome-depleted regions and in near-complete loss of enhancer-promoter interactions (Figures 5A and S5F). At other enhancers, promoter contacts were not dependent on SOX2. These retained hypersensitivity by ATAC-seq^43^ on SOX2 removal, despite containing SOX2 binding sites and demonstrable binding of SOX2 by ChIP-seq. This was a generalized phenomenon; when contacts between promoters and SOX2-bound enhancers decreased, there was loss of ATAC-seq at the enhancer, and conversely, when contacts were unchanged, there was no change in ATAC-seq signal (Figure 5C).

MCCu from SOX2-bound enhancers confirmed that loss of ATAC-seq signal correlated with dramatic changes in long-range contacts and dissipation of the adjacent nanoscale domains (Figures 5D and 5F). In contrast, subtle changes in long-range contacts and nanoscale domains were observed at enhancers at which ATAC-seq did not change substantially on SOX2 depletion. However, at these sites, the short-range contacts within the nucleosome-depleted regions changed on SOX2 depletion (Figures 5E and 5G).

NANOG depletion, using the FKBP-dTAG-13 system, had a much less profound impact on enhancer-promoter contacts (Figures 5H, 5I, and S5F). This is in keeping with previous studies, which have shown that depletion of NANOG has less effects on transcription than SOX2 depletion.^41^

Overall, we found that enhancer-promoter contacts reduced markedly when transcription factor binding was critical for maintenance of nucleosome-depleted regions. However, only small effects on large-scale structures occur when removal of the transcription factor did not result in loss of the nucleosome-depleted region. This led us to postulate that nucleosome depletion *per se* plays a critical role in mediating enhancer-promoter contacts.

### Nanoscale domains can be replicated using molecular dynamics simulations

To understand the role that nucleosomes play in establishing chromatin structure, we investigated their biophysical properties *in silico*. We performed molecular dynamics simulations of a chemically specific coarse-grained chromatin model, in which each DNA base pair is represented with an ellipsoid and every amino acid in the histone proteins is depicted with a spherical bead (Figure S6A).^44^ The model captures the fundamental physicochemical features of the nucleosomes, including sequence-dependent DNA mechanics; the secondary structure of the histone core; the flexibility of histone tails; and the size, charge, and hydrophobicity of amino acids and DNA. Notably, this approach also accounts for the natural plasticity of nucleosomes—such as breathing and sliding—by explicitly modeling the dynamic wrapping of nucleosomal DNA around the histone core. Wrapping of nucleosomal DNA around the histone core in the model is determined by the energetic balance between histone-DNA associative interactions and the mechanical deformation of DNA. As a result, the model accurately predicts chromatin structural and dynamic behavior, as a function of intrinsic factors like DNA linker length, sequence, histone composition, and nucleosome plasticity. Using this approach, we probed the structural behavior of 16-nucleosome arrays with 3,040 bp of DNA, which corresponds to ~24,700 beads in the coarse-grained model.

**Figure 6:**
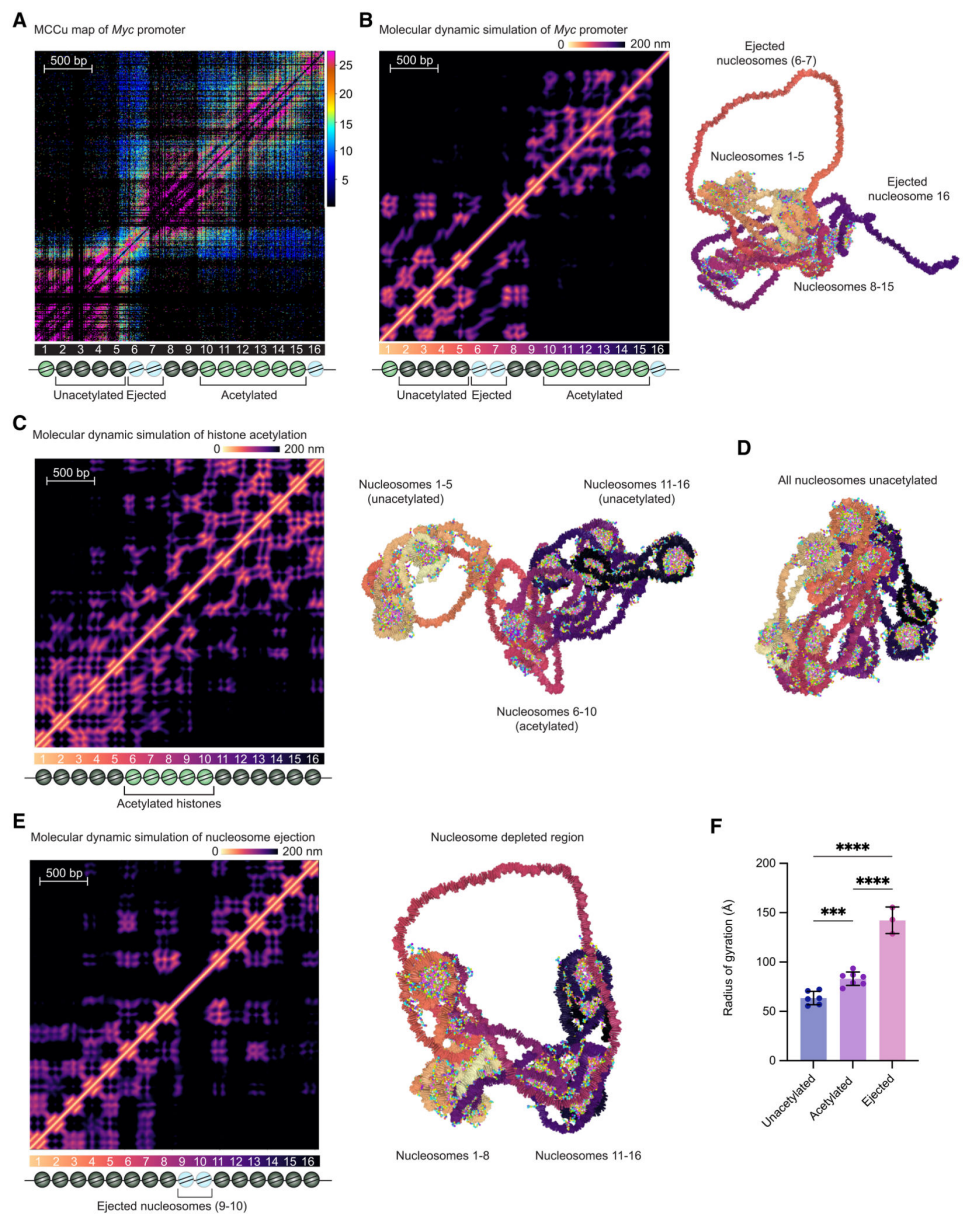
Chemically specific coarse-grained molecular dynamics simulations of the biophysical properties of chromatin recapitulates main features of 3C contact maps (A) Experimental MCCu heatmap from the *Myc* promoter with annotation of nucleosome positions and acetylation status represented below (ICE normalized read density). (B) Molecular dynamics simulation derived heatmap and representative structure of the *Myc* promoter. The DNA sequence, lysine acetylation (nucleosomes 1 and 10–15), nucleosome repeat length, and nucleosome-depleted sites (nucleosomes 6, 7, and 16) were determined from experimental data. The charge on 50% of lysine residues of H2A, H2B, H3, and H4 was neutralized, in keeping with previous nuclear magnetic resonance and mass spectrometry data^19,45^ (Video S1). (C) Computationally predicted heatmap and representative structure from the D-HREMD simulation of histone acetylation. The charge on 50% of lysine residues of H2A, H2B, H3, and H4 was neutralized^19,45^ on nucleosomes 6–10 (Video S2). (D) Computationally predicted chromatin structure from the D-HREMD simulation of 16-nucleosome arrays with no modifications to the histones (Video S3). (E) Computationally predicted heatmap and representative structure from the simulation of eviction of nucleosomes 9 and 10 (Video S4). (F) Mean radius of gyration of the different classes of nucleosomes in the simulation of the *Myc* promoter. ***adjusted *p* = 0.0008, ****adjusted *p* < 0.0001 (one-way ANOVA). Each simulation trajectory was run for 20–30 million timesteps each of 40 fs. The average of data for the final 30% of the simulation is represented in the heatmaps. See also Figure S6.

We initially aimed to determine whether this approach could accurately reproduce the high-resolution heatmaps for our genes of interest. To test this, we performed Debye-length Hamiltonian replica exchange molecular dynamics (D-HREMD) simulations^44^ of the *Myc* promoter (Figure 6A), incorporating its characteristic DNA sequence, nucleosome positions, nucleosome-depleted regions, and histone acetylation profile (Figure 6B; Video S1). D-HREMD is an advanced sampling technique required to probe the equilibrium ensemble of chromatin, given its characteristic complex, rugged energy landscape.^44^ To generate computational heatmaps, we calculated the distance between DNA ellipsoid pairs, using the data from the simulation trajectory post-equilibration at physiological salt conditions (Debye length of 8 Å), obtained after demultiplexing the set of D-HREMD replicas. The computationally predicted heatmap (Figure 6B) showed remarkable correlation with the MCCu data (Figure 6A).

To better understand the relative effects of histone acetylation and nucleosome ejection, we investigated the effects of altering these properties sequentially. Initially, we compared D-HREMD simulations of histones with acetylation of 50% of the histone tail lysines in the central 5 nucleosomes (Figure 6C; Video S2) with a control 16-nucleosome array, with no modifications (Figure 6D; Video S3). Previous work has shown that acetylation of histone tails alters the biophysical properties of chromatin.^20^ Acetylation leads to neutralization of the positive charge on lysine residues, which reduces the electrostatic interactions with the phosphate groups in the DNA backbone. This effect was also observed in our experimental data, which showed that H3K27ac correlated with more relaxed nucleosome positioning (Figure S2G). Our D-HREMD simulations showed that histone acetylation alters the biophysical properties of nucleosomes and that this results in domain formation (Figure 6C). In addition, we observed that histone acetylation results in increased nucleosome breathing. This manifests as less distinct nucleosome positioning signals in the heatmaps of the mean distances between DNA bases computed from the simulations (Figure 6C).

We next investigated the effects of nucleosome eviction by conducting an additional set of D-HREMD simulations where nucleosomes 9 and 10 were removed. Our results revealed that nucleosome eviction creates a long, highly flexible segment of free DNA, which significantly reduces the attractive forces between the flanking nucleosomes. This reduction in nucleosome attraction causes chromatin to partition into two distinct globules (Figure 6E; Video S4), which correlate closely with the nanoscale domains observed in our experimental data. Analysis of the equilibrium ensembles of chromatin obtained from our simulation revealed that the flexibility and structural heterogeneity of chromatin, as indicated by the fluctuations and mean values of the radius of gyration, increased following histone acetylation and were further amplified after nucleosome eviction (Figures 6 and S6; Video S5).

Due to its overall negative charge, chromatin condenses only when nucleosome-nucleosome interactions and the stabilizing effects of counterions in solution outweigh the strong electrostatic repulsion between the DNA linkers. Our molecular dynamics analysis confirms that at physiological salt concentrations, nucleosome-nucleosome interactions are highly sensitive to modifications in the positively charged lysine residues on the histone tails, which play a key role in regulating chromatin compaction. We also observed that the removal of histones introduces long, nucleosome-depleted DNA segments that exhibit increased flexibility stemming from a significantly higher total negative charge and a notable increase in electrostatic self-repulsion. Therefore, the two nucleosomes flanking the nucleosome-depleted region present a significantly weakened capacity to form electrostatic associations with other nucleosomes. This reduction in their attractive forces leads to the separation of chromatin regions on either side of the nucleosome-depleted site. Based on this observation, we propose that the regions between nucleosome-depleted sites tend to cluster together, forming nanoscale chromatin domains, which are visible in our high-resolution heatmaps (Figure 3D).

## Discussion

Here, we have generated multidimensional 3C data at single base-pair resolution, enabling chromatin structure to be resolved in unprecedented detail. We have visualized complex structures within nucleosome-depleted regions at active regulatory elements on the scale of individual transcription factor binding sites. The high resolution of MCCu allowed for the integration of 3C data with chemically specific molecular dynamics simulations of chromatin. This has led us to propose an initial unified model based on the integration of data from the orthogonal approaches, which we outline below.

Our simulations and other studies show stochastic folding of chromatin, which retains liquid like behavior,^44,46^ consistent with phase separation models,^28,29,47–49^ but the central condensed core of unacetylated nucleosomes may form a solid hydrogel as previously suggested.^50^ We believe our data are also consistent with 3D super-resolution imaging and electron microscopy studies. These have shown that chromatin forms irregularly shaped structures around 200–300 nm in size, which assemble to form a sponge-like structure on a nuclear scale (Figure S7A).^51–53^ Between chromatin dense structures resides an interchromatin compartment containing RNA, which forms a complex network of channels that transport macromolecules to the nuclear pores.^51–53^ The chromatin compartment itself segregates into domains with different functional properties and levels of chromatin compaction. RNA polymerase II, histone modifications associated with active genes (H3K4me3/H3K36me3), and the cohesin complex localize to the interchromatin space and adjacent perichromatin compartment. In contrast, polycomb silenced genes, and heterochromatin are positioned in the dense interior chromatin compartment^52^ (Figure S7A).

**Figure 7:**
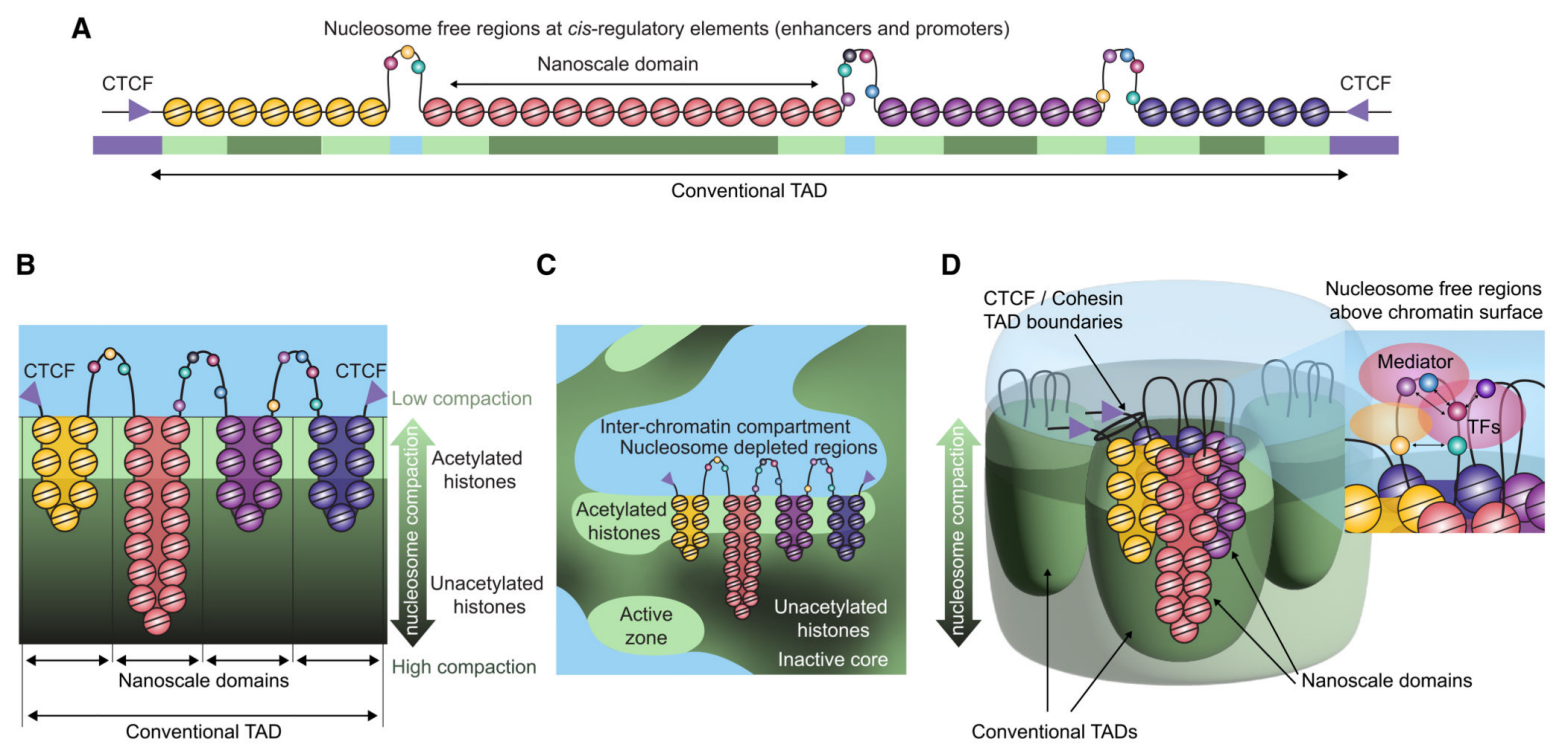
Proposed model of enhancer-promoter contacts (A) Linear genome annotation depicting a simplified regulatory region, consisting of CTCF boundary elements, nucleosome-depleted regions (corresponding to *cis*-regulatory elements), and regions of adjacent histone acetylation. (B) Folding of the linear genome driven by nucleosome condensation, which separates the nucleosome-depleted regions from an acetylated layer and a central core of highly compacted nucleosomes. The nucleosome-depleted regions potentially coalesce on or above the surface of the aggregated nucleosomes. (C) Overlay of the model onto a nuclear image showing how the nucleosome-depleted regions potentially coalesce in the interchromatin compartment and how histone acetylation might lead to compartmentalization adjacent to the interchromatin compartment. The interchromatin compartment contains high levels of RNA polymerase. (D) Cohesin-mediated loop extrusion leads to strong interactions between the boundary CTCF sites. This leads to the formation of conventional TADs, which leads to isolation of rafts of nucleosomes from surrounding regions. Within these CTCF-bound regulatory domains, nucleosome-depleted regions coalesce above the surface of the nucleosome containing compartment. See also Figure S7.

Many loci contain multiple nucleosome-depleted enhancers and promoters, which are commonly surrounded by regions of histone acetylation (Figure S7B) and bound by convergent CTCF sites to form TADs (Figure 7A). The MCCu data and molecular dynamics simulations align with existing studies,^54^ which show that the default state for chromatin is one of nucleosome condensation, in which nucleosomes largely only make local contacts. This is consistent with super-resolution studies showing the presence of an inner core of nucleosomes, where transcription is likely repressed through exclusion of large molecular complexes such as polymerase.^52,55^

Our data are also in keeping with decades of previous work,^20,56^ which shows that acetylation leads to a more open and transcriptionally active chromatin conformation. The molecular dynamics simulations show that neutralization of positive charges on acetylated lysine groups reduces the affinity of histone proteins for the negatively charged backbone of DNA, and this per se reduces chromatin compaction. This aligns with *in vitro* work showing that acetylation diminishes the propensity of histone tails to drive phase separation.^57^ In addition, the molecular dynamics simulations show that acetylated chromatin regions tend to separate from denser unacetylated regions. We propose that differences in the biophysical properties of nucleosome proteins and DNA lead to self-assembly of the linear polymer structure into nanoscale domains, which are ordered by histone acetylation (Figure 7D). To link the biophysical effects of histone acetylation with previous imaging studies, we undertook super-resolution imaging of H3K27ac. This showed that regions of histone acetylation form discrete foci that highly correlate with areas of lower chromatin density, which are juxta-posed with the interchromatin space (Figure S7C).

Histone acetyltransferases (such as KAT2A/2B, KAT6A/B, and KAT7) are recruited at active enhancers, and these acetylate the nucleosomes adjacent to the nucleosome-depleted region.^58^ We propose that acetylation helps to stabilize enhancers near the interface between the chromatin and interchromatin compartments through alterations in the biophysical properties of the nucleosomes.

It is well established that some transcription factors access DNA in compacted chromatin.^59,60^ It has recently been demonstrated by cryo-electron microscopy that transcription factors can cause changes in nucleosome structure, including repositioning of nucleosomal DNA, which facilitate co-operative binding of other transcription factors.^61^ The release of DNA from nucleosomes increases the length of the nucleosome-depleted section (146 bp ~11 nm on the nucleosome compared with ~50 nm in the free state). This increases the flexibility of the nucleosome-depleted section, and our data and other studies in *S. cerevisiae*^54,62^ have shown that this leads to partitioning of the nucleosomes on either side into nanoscale self-associating domains. The increase in size of active enhancers and promoters leads to an increased area for physical proximity and may in part explain the paradox that imaging studies show that the volume occupied by these elements expands on activation,^4,6,63,64^ and yet 3C studies show that they come into closer proximity.^7,65–67^

We find that highly localized 3C signals occur between adjacent nucleosome-depleted regions, and we observe that signals between individual regulatory elements are generally non-specific, with all nucleosome-depleted regulatory elements within a domain contacting one another. An exception to this is CTCF binding sites, which we observed to contact one another specifically, in keeping with previous 3C studies.^4,7,68^

This led us to hypothesize that nucleosome-depleted regions coalesce above the surface of the acetylated nucleosomes in the perichromatin layer or interchromatin compartment (Figures 7C and 7D). This potentially results in accessibility of nucleosome-depleted regions to the interchromatin and perichromatin compartments, containing RNA polymerase II and large protein complexes, such as Mediator and the PIC.

We find that when depletion of SOX2 leads to dissipation of nucleosome-depleted regions, physical contacts disappear with other nucleosome-depleted regulatory elements in the vicinity. Combined with our molecular dynamics simulations, this leads us to conclude that nucleosome removal *per se* alters the biophysical properties of chromatin and drives contacts between adjacent nucleosome-depleted regions. We hypothesize that the localized proximity signal between adjacent nucleosome-depleted regions results from restriction of the diffusion of nucleosome-depleted regions to two dimensions by the chromatin surface, which markedly increases the probability of physical contacts. The chromatin surface may provide an area for the large protein complexes comprising the PIC and Mediator to assemble.

Within nucleosome-depleted regions, we observed highly specific contacts between transcription factor binding sites, and our observations are consistent with nucleosome-depleted regions forming specific structures driven by transcription factor binding. Small-scale contacts were dependent on binding of Mediator, and they likely serve as a foundation for the assembly of the large protein complexes required for transcription. These large protein complexes may prevent diffusion of regulatory elements back into the nucleosome dense compartment and stabilize the nucleosome-depleted region on or above the surface of the nucleosome compartment.^69^

Our data also support the current models of loop extrusion.^26,27^ Many studies have shown that loop extrusion results in strong insulating contacts between convergent CTCF binding sites at TAD boundaries. In this model, we hypothesize that this leads to the formation of large rafts of chromatin containing multiple nanoscale domains (correlating with conventional TADs), which are held together by CTCF and cohesin (Figure 7D). We and others have found that the cohesin loading protein NIPBL is principally located by ChIP-seq at active enhancers and promoters.^7,70^ In addition, microscopy studies have shown that CTCF and cohesin localize to the interchromatin and perichromatin compartments.^52^ Thus, loading of cohesin and potentially loop extrusion may occur at the surface of the nucleosome compartment. The model predicts, however, that on a small scale the biophysical properties of the nucleosomes play a more important role in determining chromatin structure than cohesin; potentially explaining the relatively subtle changes in transcription and enhancer-promoter contacts seen on cohesin depletion.^31,68,71–75^

Our high-resolution MCCu data combined with molecular dynamics simulations and super-resolution microscopy have allowed us to propose a model that unifies the data from orthogonal approaches. We hypothesize that regulatory elements make physical contacts above a condensed nucleosome surface. This may provide a mechanism for the integration of signals from multiple regulatory elements, which is critical for the evolution of the complex patterns of tissue-specific gene regulation seen in higher eukaryotes.

### Limitations of the study

MNase-based experiments inherently produce ligation junctions adjacent to DNA binding proteins. Although we developed a computational method to mitigate this effect, some sequences responsible for small-scale contacts may not be precisely identified. Our experimental design is restricted to 15 gene promoters and 25 enhancers, all of which representing only a fraction of the genome, and the findings may not be fully generalizable across all genomic contexts. Our perturbation experiments using degron systems were limited to five proteins, and although we used a combination of techniques to show marked protein degradation following dTAG-13 treatment, it is possible that small amounts of residual chromatin-bound protein could confound results. While these experiments provide valuable mechanistic insights, further studies examining a broader panel of targeted proteins would be necessary to draw comprehensive conclusions about protein-specific effects on chromatin architecture.

## Resource Availability

### Lead contact

Requests for further information and resources should be directed to and will be fulfilled by the lead contact, James Davies (james.davies@imm.ox.ac.uk).

### Materials availability

All unique/stable reagents generated in this study are available from the lead contact with a completed materials transfer agreement.

## Star★Methods

### Key Resources Table

**Table:** 

REAGENT or RESOURCE	SOURCE	IDENTIFIER
Antibodies		
ter119-phycoerythrin antibodies	Biolegend	cat# 116208; AB_313709
Anti-phycoerythrin beads	Miltenyi Biotec	cat# 130-048-801; AB_244373
Rabbit anti H3K27ac	Cell Signalling	cat# D5E4: AB_10949503
Mouse anti H3K27me3	Abcam	cat# ab6002; AB_305327
Donkey anti rabbit AF488	Invitrogen	cat# A11-008; AB_143165
Donkey anti mouse AF555	Invitrogen	cat# A-31570; AB_2536180
MED13L Polyclonal Antibody	Thermo Fisher	cat# A302-420A; AB_1907303
T7-Tag (D9E1X) XP ® Rabbit mAb	Cell Signaling	cat# 13246S: AB_2798161
Anti-HA tag antibody	Abcam	cat# ab9110; AB_307019
Anti-HA.11	BioLegend	cat# MMS-101R(16B12); AB_291262
Anti-SNRP70/U1-70K antibody	Abcam	cat# ab316762: AB_2193699
Anti-Histone H3 antibody	Abcam	cat# ab1791; AB_302613
Vinculin antibodies	Abcam	cat# Ab129002; AB_11144129
Biological samples		
Mouse erythroid cells	this paper	C57BL/6
Mouse haemopoietic stem cells	this paper	C57BL/6
Chemicals, peptides, and recombinant proteins		
FCS used in ES cell cultures	Gibco	cat# 10270-106
FBS used in RPE1 culture	Sigma	cat# F7542
Gelatin	Sigma	cat# 900628-1G
Glutamine	Gibco	cat# 25030-024
GlutaMAX	Gibco	cat# 35050061
Sodium pyruvate	Gibco	cat# 11360-039
Non-essential amino acids	Gibco	cat# 11140-035
Mercaptoethanol	Gibco	cat# 31350-010
Penicillin-streptomycin	Gibco	15140122
LIF	Thermo Fisher	cat# PMC9484
Formaldehyde	Sigma	cat# 47608-250ML
Digitonin	Sigma	cat# D-141
ethylene glycol-bis(2-aminoethylether)- N,N,N’,N’-tetraacetic acid (EGTA)	Sigma	cat# E3889
dATP, dCTP, dGTP and dTTP	Thermo Fisher	cat# R0191
MNase	NEB	cat# M0247
T4 Polynucleotide Kinase PNK	NEB	cat# M0201L
DNA Polymerase I Large (Klenow) Fragment	NEB	cat# M0210L
T4 DNA ligase	Thermo Fisher	cat# EL0013
DNeasy blood and tissue kit	Qiagen	cat# 69506
Ampure XP beads	Beckman Coulter	cat# A63881
NEB Ultra II	NEB	cat# 7645
HyperCapture Target Enrichment Kit	Roche	cat# 9075828001
Streptavidin beads	Thermo Fisher	cat# M270
Gibson Assembly Master Mix kit	NEB	cat# E2611
Lipofectamine 3000	Thermo Fisher	cat# L3000015
Trans-Blo Turbo Mini Polyvinylidene fluoride (PVDF) Transfer Packs	Biorad	cat# 1704156
Pierce ECL Western Blotting Substrate	Thermo Fisher	cat# 32106
Di (N-succinimidyl) glutarate (DSG)	Merck	cat# 80424
Protein A Dynabeads	Thermo Fisher	10001D
Protein G Dynabeads	Thermo Fisher	10003D
ChIP DNA Clean & Concentrator kit	Zymo	cat# 5201
Igepal CA-630	Sigma	cat# 56741
EverBrite	Biotium	cat# 23001
Paraformaldehyde	Thermo Scientific	cat# 28906
DAPI	Merck	cat# 10236276001
Coverslips	Marienfeld	cat# 0117550
BlockAid	Invitrogen	cat# B10710
Critical commercial assays		
TapeStation D1000 ScreenTape	Agilent	cat# 5067-5582
TapeStation D1000 reagents	Agilent	cat# 5067-5583
TapeStation RNA ScreenTape	Agilent	cat# 5067-5576
Qubit dsDNA BR Assay Kit	Invitrogen	cat# Q32850
Qubit dsDNA HS Assay Kit	Invitrogen	cat# Q32851
Deposited data		
MCC data	This paper, Gene Expression Omnibus	GEO: GSE277286
TT-seq data	This paper, Gene Expression Omnibus	GEO: GSE281416
ATAC-seq data	This paper, Gene Expression Omnibus	GEO: GSE281414
ChIP-seq data	This paper, Gene Expression Omnibus	GEO: GSE307191
MNase-seq data	This paper, Gene Expression Omnibus	GEO: GSE307228
Experimental models: Cell lines		
Mouse embryonic stem cells	Dr A. Smith, University of Edinburgh	E14TG2a
RPE1 cells	ChEMBLdb	cat# CRL4000, RRID CVCL_4388
Experimental models: Organisms/strains		
Mus musculus	Jackson laboratories	C57BL/6
Oligonucleotides		
Biotinylated Capture Oligonucleotides (pools)	IDT	N/A
Biotinylated Capture Oligonucleotides (individual probes)	Sigma Aldrich	N/A
Recombinant DNA		
SpCas9-2A-Ruby2 (pX458)	Addgene	cat# 110164
pSptCas9(BB)-2A-Puro (PX459)	Addgene	cat# 2988
HDR donor vectors	GeneArt Gene Synthesis custom design and in-house cloning	N/A
Software and algorithms		
MCC analysis tools	https://doi.org/10.1038/s41586-021-03639-4	Oxford University Software Store https://process.innovation.ox.ac.uk/software/
MCCu analysis tools	This paper	Oxford University Software Store https://process.innovation.ox.ac.uk/software/
Lotron MCC	This paper	Oxford University Software Store https://process.innovation.ox.ac.uk/software/
Lanceotron	https://doi.org/10.1093/bioinformatics/btac525	https://lanceotron.molbiol.ox.ac.uk/
bowtie2	https://doi.org/10.1038/nmeth.1923	https://github.com/BenLangmead/bowtie2
JASPAR	https://doi.org/10.1093/nar/gkab1113	https://jaspar.genereg.net/
DESeq2	https://doi.org/10.1186/s13059-014-0550-8	http://www.bioconductor.org/packages/release/bioc/html/DESeq2.html
bedtools	https://doi.org/10.1093/bioinformatics/btq033	https://bedtools.readthedocs.io/en/latest/
Trim_galore	Babraham Institute	https://www.bioinformatics.babraham.ac.uk/projects/trim_galore/
FLASh	https://doi.org/10.1093/bioinformatics/btr507	John Hopkins Centre for Computational Biology https://ccb.jhu.edu/software/FLASH/
LAMMPS	https://doi.org/10.1016/j.cpc.2021.108171	https://www.lammps.org
OVITO	https://doi.org/10.1088/0965-0393/18/1/015012	https://www.ovito.org
SoftWoRx	GE Healthcare	Available from Cytiva
SIMCheck^83^	https://doi.org/10.1038/srep15915	https://www.micron.ox.ac.uk/software/SIMcheck.php
FIJI^84^	https://doi.org/10.1038/nmeth.2019	https://imagej.net/software/fiji/
MorphoLibJ^85^	doi: 10.1093/bioinformatics/btw413.	https://imagej.net/plugins/morpholibj
Chromagnon	https://doi.org/10.1038/s41598-018-25922-7	https://github.com/macronucleus/Chromagnon
CLIJ^84^	https://doi.org/10.1038/s41592-019-0650-1	https://clij.github.io/

### Experimental Model And Study Participant Details

#### Preparation of murine erythroid cells

This research was conducted under the project license PP3929317, adhering to the European Union directive 2010/63/EU and the UK Animals (Scientific Procedures) Act of 1986. All procedures were reviewed by the Animal Welfare and Ethical Review Body of the Department of Biomedical Services at the University of Oxford. Murine erythroid cells were purified using Ter119 selection (ter119-phycoerythrin antibodies (Biolegend 116208) and anti-PE MACS beads (Miltenyi, 130-048-801)) from the spleen of C57BL/6J adult mice including both sexes following administration of phenylyhydrazine (40 mg/g body weight, 3 doses, 12 h apart). Murine erythroid cells were also collected from fetal livers dissected from E12.5 embryos of C57BL/6J mice including both sexes. Dissociated cell suspension was then expanded for 4 days in Stempro (Invitrogen) supplemented with erythropoietin (1U/ml), mSCF (50ng/ml) and dexamethasone (0.4 μg/mL).

#### Preparation of murine HSPCs

Mouse hematopoietic stem and progenitor cells (HSPCs) were extracted and cultured as previously described.^86^ Briefly, 8-12-week-old mice including both sexes were euthanized under terminal anaesthesia by cervical dislocation in accordance with Schedule 1 guidelines. The femurs and tibias were aseptically harvested, and the marrow cavities were mechanically disrupted using a pre-chilled mortar and pestle. The resulting cell suspension was passed through a 50 μm nylon strainer (Sysmex Partec 04-0042-2317) to remove debris. Total bone marrow cells were incubated with APC-conjugated anti-CD117 antibody (eBioscience) at 4 °C for 30 minutes, followed by two washes in PBS. Cells were then labelled with anti-APC magnetic microbeads (Miltenyi Biotec 130-090-855) for 20 minutes at 4 °C and washed once more in PBS. CD117^+^ cells were enriched on LS columns per the manufacturer’s instructions (Miltenyi Biotec 130-042-401). Purified cells were cultured in Ham’s F-12 Nutrient Mix (Gibco) supplemented with 10 mM HEPES (Gibco), 1 mg/mL polyvinyl alcohol (PVA), 1× penicillin-streptomycin-glutamine (Gibco), 100 ng/mL murine thrombo-poietin (TPO; Miltenyi Biotec), and 10 ng/mL murine stem cell factor (SCF; PeproTech). Cultures were maintained in 24-well Corning CellBIND plates (Corning 3337) at 37 °C with 5% CO_2_ for 7–14 days prior to downstream applications.

#### Cell lines

The murine embryonic stem cell line (E14TG2a, male biological origin) were maintained in 0.1% diluted gelatin (Sigma, 900628-1G) coated flasks in Glasgow MEM x1 liquid (Gibco 21710-025) with 10% FCS (Gibco 10270-106), 1x Glutamine (Gibco 25030-024), 1 mM sodium pyruvate (Gibco 11360-039), 1x non-essential amino acids (Gibco 11140-035), 100 μM mercaptoethanol (Gibco 31350-010) and 10 ng/mL LIF (Thermo Fisher PMC9484).

The hTERT-immortalized human retinal pigment epithelial cells (RPE1, female biological origin) were maintained in Dulbecco’s Modified Eagle’s Medium (Gibco 11960044) supplemented with 10% fetal bovine serum (Sigma, F7524), 1% GlutaMAX (Gibco, 35050061), 1% non-essential amino acids, and 1% penicillin-streptomycin (Gibco, 15070063) in a humidified incubator at 37°C with 5% CO_2_.

### Method Details

#### 3C library manufacture

Aliquots of 1 x 10^7^ cells were fixed in Formaldehyde, 37% (vol/vol) (Sigma, 47608-250ML) at a final concentration 2% for 10 minutes at room temperature with constant rotation. After 10 minutes, reaction was quenched with 1 M glycine to a final concentration 130 mM and centrifuged (5 min, 300 g). The supernatant was removed, and the cell pellet was washed with cold PBS and reconstituted in 1 mL PBS. Cells were permeabilised with Digitonin (Sigma D-141; final concentration of 0.005% wt/vol). Cells were split into 1 mL aliquots, each with 10–20 million cells, and snap frozen and stored at -80 °C. Subsequently, defrosted, permeabilised cells were centrifuged (5 min, 300 g) the supernatant discarded, and resuspended in 890 μL PCR-grade water. Every aliquot of fixed cells were split into 3 separate digests to allow titration of MNase (NEB M0247) concentration, which typically ranged from 5 to 20 Kunitz units per reaction. Each digestion reactions was prepared with a variable quantity of PCR-grade water and MNase, along with 80 μL of reduced calcium content MNase buffer (Tris-HCl pH 7.5 10 mM; CaCl_2_ 1 mM). Reactions were incubated for 1 hour at 37 °C in an Eppendorf Thermomixer at 500 rpm and quenched by adding ethylene glycol-bis(2-aminoethylether)-N,N,N’,N’-tetraacetic acid (EGTA) (Sigma E3889) to a final concentration of 5 mM. Samples were then centrifuged (5 min, 300 g), the supernatant was removed, and the pellets were resuspended in 1 mL PBS with 10 μL EGTA. To evaluate digestion efficiency, 20% of the reaction was removed. The remaining reaction was centrifuged (5 min, 300 g) and the supernatant containing digestion buffer was discarded. The cell pellet was washed in PBS and the cells resuspended in DNA ligase buffer (Thermo Scientific; final concentrations Tris-HCl pH 7.5 40 mM, MgCl_2_ 10 mM, DTT 10 mM, 5 mM ATP) supplemented with dNTPs (final concentration 400 μM of each of dATP, dCTP, dGTP and dTTP (Thermo Fisher R0191)) and EGTA 5 mM.

The reaction mixture was then treated with T4 Polynucleotide Kinase PNK (NEB M0201L) and DNA Polymerase I Large (Klenow) Fragment (NEB M0210L) at final concentrations of 200 U/ml, and 100 U/ml respectively, T4 DNA ligase (Thermo Scientific, High Concentration Ligase (30 U/μL) EL0013) was added to a final concentration of 300 U/ml and the reaction was incubated at 37°C for 2 hours and then 20°C overnight using an Eppendorf Thermomixer at 500 rpm. Chromatin was decrosslinked (proteinase K, 65 °C, >2 hours) and DNA was purified using the Qiagen DNeasy blood and tissue kit (Qiagen 69506). Digestion and ligation efficiencies were assessed using the Agilent Tapestation (D1000 reagents).

#### Sequencing library preparation

Library preparation was performed as previously described.^7,87^ Initially, 2-6 μg of each 3C library was sonicated to an average fragment size of 200 bp using a Covaris S220 Focused Ultrasonicator (3 cycles of 60 seconds, duty cycle 10%, intensity 5, 200 cycles per burst). After sonication, the size of library was evaluated using an Agilent TapeStation (DNA 1000). The sample was then cleaned using Ampure XP beads (Beckman Coulter A63881). To maximize complexity, the fragmented material was divided into two independent library preparation reactions. Library construction followed the NEBNext Ultra II protocol (NEB, 7645S) with minor adjustments: 5 μL of adaptor was used in the ligation step, bead clean-ups were performed at 1.8×, and final amplification was carried out in duplicate using Herculase II polymerase (Agilent 600677) to improve DNA yield.

#### Capture oligonucleotide design

Capture oligonucleotides of 120 bp were designed using the CapSequm tool (https://github.com/jbkerry/capsequm). These were tiled with a 50% overlap to the regions of interest. See Table S1 for details of the capture oligonucleotide used.

#### Biotinized oligonucleotide production

The biotinylated oligonucleotide was generated by asymmetric PCR. Initially, a 120-bp oligonucleotide was designed to target the sequence of interest using CapSequm tool (https://github.com/jbkerry/capsequm). The design involved generating the reverse complement of the target oligo and the reverse complement of a 15 bp M13 forward primer (Biotin-GTTTTCCCAGTCACG) to serve as the binding sequence; this binding sequence was then appended to the 3’ end of the reverse complemented oligo to yield a full 135 bp template. PCR amplification was performed using Q5 PCR Master Mix (25 μL) with the oligo pool at 1 μM and a biotinylated primer at 100 μM, making up a final volume of 50 μL with water. The cycling conditions included an initial denaturation at 98°C for 30 sec, followed by 30–35 cycles of 98°C for 10 sec, annealing at 57°C (depends on primer Tm) for 30 sec, extension at 72°C for 20 sec, and a final extension at 72°C for 2 min, with a final hold at 4°C. PCR clean-up was carried out using the Oligo Clean & Concentrator kit (Cat. No.: D4060).

#### Oligonucleotide hybridisation, capture and sequencing

For target enrichment, 1–2 μg from each of the indexed capture libraries were combined to generate a pooled sample of 12 μg per capture reaction. Hybridization was performed using the Roche HyperCapture Target Enrichment Kit (cat. no. 9075828001) with modifications from the manufacturer’s instructions.^87^ 10 μL of species-specific COT DNA were added per library. This mixture was denatured by heating to 95°C for 10 minutes before hybridizing with 120 base-pair biotinylated oligonucleotides pool at a total concentration ranging from 29 nM to 2.9 μM. To improve capture efficiencies for high resolution visualisation of enhancer–promoter contacts, oligonucleotides were either individually synthesised (Sigma-Aldrich) or prepared in house to allow higher concentrations of oligonucleotides to be used (2.9 μM pools). The hybridization was carried out for 72 h using Roche HyperCapture Target Enrichment Kit reagents. Post-hybridization, the samples were captured with streptavidin beads (Thermo Fisher M270), followed by washing and amplification according to Roche HyperCapture standard protocols. A second round of oligonucleotide capture was conducted with the same oligonucleotides pool and reagents at adjusted volume and a 24 h hybridisation time. Sequencing was performed on the Illumina platform with 300 bp reads, utilizing paired-end 150 bp reads.

#### Genetic engineering of ES cells

The FKBP12^F36V^ sequence was engineered homozygously into the endogenous loci using CRISPR-Cas9.^41,88^ For mediator degrons, selected sgRNAs were designed and cloned into the pX458-Ruby backbone and the FKBP12^F36V^ sequence was synthesized by GeneArt (Thermo Fisher Scientific). Gibson assembly was used to construct the targeting construct (Gibson Assembly Master Mix, NEB) from the PCR-amplified synthetic FKBP12^F36V^ sequence and 450-800 bp homology arms surrounding the start or stop codon of the target gene, which were amplified from the mouse genomic DNA. The targeting construct was designed such that the Cas9 recognition site is disrupted by the insertion of the tag. Mouse ES cells were transfected in a single well of a 6-well plate with 0.5 μg Cas9 guide plasmids (SpCas9-2A-Ruby2 (pX458 Addgene 110164) and pSptCas9(BB)-2A-Puro (PX459 Addgene 2988)) and 1-2 μg targeting construct plasmid using Lipofectamine 3000 (Thermo Fisher L3000015) according to the manufacturer’s guidelines. The day after transfection, cells were subjected to mRuby fluorescent sorting selection or puromycin selection and passaged at a range of densities. Approximately 5-7 days following transfection, individual clones were isolated, expanded and PCR-screened for the homozygous presence of the tag. For the transcription degrons, the FKBP sequence was engineered and the knock-in was generated as previously described.^41^

#### Western Blot

Cells were collected, washed once with PBS, and heated in freshly made SDS loading buffer (50 mM Tris-HCl (pH 6.8); 100 mM Dithio-threitol DTT; 2% Sodium Dodecyl Sulfate (SDS); 10% glycerol; 0.05% Butterfields Phosphate Buffer (BPB), 0.2% β-mercaptoethanol) to 95°C for 20 min. Lysates were separated on 10% SDS-PAGE gels in 1 × running buffer (2.5 mM Tris Base, 19.2 mM Glycine, 0.01% SDS in H_2_O, pH 8.3). Proteins were then transferred onto a Polyvinylidene fluoride (PVDF) membrane (Millipore) in 1 × transferring buffer (1.2 mM Tris Base, 9.6 mM Glycine, pH 8.3). After transfer, the membrane was blocked with 4% blocking buffer (Bio-Rad) for at least 30 min at RT with shaking. The membrane was incubated with the primary antibody in blocking buffer overnight at 4 °C with shaking. The next day, after three washes with PBST (PBS with 0.05% Tween-20), the membrane was incubated with the secondary antibody in blocking buffer for 1 hour at RT with shaking and washed three times in PBST. Finally, the membrane was illuminated using Pierce ECL Western Blotting Substrate (Pierce Endogen) according to the manufacturer’s instructions. Subcellular fractionation was carried out by lysing the cell membrane with a detergent buffer (10 mM HEPES pH 7.9, 10 mM KCL, 1.5 mM MgCl_2_, 0.34 M sucrose, 10% glycerol, 0.2% (wt/vol) IGEPAL CA-630, protease inhibitor cocktail), extracting the nucleoplasm fraction with a no-salt buffer (3 mM EDTA, 0.2 mM EGTA, protease inhibitor cocktail) and the chromatin-bound fraction with a high salt buffer (20 mM Tris-HCl pH 8.0, 300 mM KCl, 5 mM EDTA, 20% glycerol, 0.5% IGEPAL CA-630, protease inhibitor cocktail). Aliquots from each fraction were collected and loaded in equal amounts. Denatured proteins were separated using NuPAGE Tris-Acetate 7% and NuPAGE Bis-Tris 4–12% gels and transferred to poly-vinylidene fluoride membranes. Western blotting was performed as described above, with antibodies listed in the key resources table.

#### ChIP-seq

ES cells were fixed for 30 min with 2 mM Di (N-succinimidyl) glutarate (DSG) (Sigma-Aldrich, 80424) followed by 30 minutes with 1% formaldehyde. Fixed cells were lysed in SDS lysis buffer (1% SDS (Sigma-Aldrich, 436143-25G), 10 mM EDTA (Sigma-Aldrich, 688363), 50 mM Tris-HCl, pH 8 with protease inhibitor cocktail (Abcam, b271306)) and the lysate was syringe-passaged through a 27-gauge needle. The cell lysates were sonicated using Covaris ME220 for 10 min. The sonicated lysates were pre-cleared with a 1:1 mix of Protein A and Protein G Dynabeads (Thermo Fisher 10001D / 10003D) for 15 minutes at 4°C. After three washes of the Dynabeads in PBS containing 0.01% tween-20 (Sigma-Aldrich, P6585-10ML), the sonicated samples were incubated overnight at 4°C. 5% of the lysate was set aside as input. After incubation, the Dynabead mix was added to each immunoprecipitate and rotated at 4°C for 3 h. The beads were washed three times with RIPA buffer (50 mM HEPES-KOH, pH 7.6, 500 mM LiCl, 1 mM EDTA, 1% NP-40, 0.7% Na-deoxycholate) and once with TE-50 mM NaCl. The chromatin was eluted from the beads with SDS lysis buffer for 30 min at 65 °C, then treated with 1μL 500 ug/ml RNase A (Invitrogen, AM2286) for 30 min at 37 °C, followed by Proteinase K (Thermo Fisher, EO0491) 1μL 14-22 mg/ml overnight at 65 °C. DNA was purified using the ChIP DNA Clean & Concentrator kit (Zymo 5201). Library preparation for the Illumina platform was performed following the manufacturer’s instructions (NEB, 7645S).

#### Transient Transcriptome RNA-Sequencing

For each of the three replicates, 5x10^7^ cells were incubated with 500 μM 4-thiouridine (Enzo/Jena Bioscience, NU-1156S) for 5 minutes, followed by pelleting and lysis in Trizol (Thermofisher, 15596026). To each sample, 60 ng of spike-in RNA was added, consisting of three transcripts labeled with 4-thiouridine from a CAS9-encoding vector (Amplified from pX458). The total RNA was extracted, treated with TURBO DNase (Thermo fisher, AM1907), and subjected to sonication for 10 seconds. The RNA was then labeled with EZ-Link HPDP-Biotin (Pierce, 21341) for 90 minutes and purified via chloroform/isoamyl alcohol extraction. The labeled nascent RNA was captured using the uMACS Streptavidin Kit (Miltenyi, 130-074-101) as per the manufacturer’s protocol, using 200 μg of input RNA. RNA was further purified with the RNeasy MinElute Cleanup Kit (Qiagen, 74204). Libraries were sequenced using 80 bp paired-end sequencing on a NextSeq500.

#### ATAC-sequencing

A total of 150,000 cells were lysed in 50 μL of ice-cold lysis buffer (10 mM Tris-HCl pH 7.4, 10 mM NaCl, 3 mM MgCl_2_, 0.1% IGEPAL CA-630) and centrifuged at 500 g for 10 minutes at 4 °C. The resulting nuclear pellet was resuspended in transposase reaction mix (1× Illumina Tagment DNA Buffer, 20034197) containing 2.5 μL of Tn5 transposase and incubated at 37 °C for 30 minutes. Transposed DNA was purified using the MinElute PCR Purification Kit (Qiagen, 28004) according to the manufacturer’s protocol, and sub-sequently PCR-amplified with Nextera index primers and NEBNext Ultra II Q5 Master Mix (NEB, M0544S) for 12 cycles. The amplified libraries were cleaned with the MinElute PCR Purification Kit and sequenced using 75 bp paired-end reads.

#### MNase-sequencing

MNase digested samples were sequenced by ligating sequencing adaptors using the NEB Ultra II reagents (NEB, 7645S) without a size-selection step. The samples were sequenced using Illumina-based sequencing with 300-bp reads (150-bp paired-end reads).

#### Imaging of Histone Modifications

RPE1 cells were grown on coverslips (Marienfeld, 0117550) overnight. The cells were fixed with 3% paraformaldehyde (Thermo Scientific, 289060+ 0.1% triton for 15 minutes at 37°C. Coverslips were blocked with BlockAid (Invitrogen, B10710) for 30 minutes and then incubated with 1:100 rabbit H3K27ac antibody (Cell Signalling, D5E4) and 1:200 mouse H3K27me3 antibody (Abcam, ab6002) for 1 hour, followed by 1:1000 donkey anti rabbit AF488 (Invitrogen, A-21206) and 1:1000 donkey anti mouse AF555 (Invitrogen, A-31570) for 1 hour. The coverslips were counterstained with 0.5 μg/mL DAPI (Merck, 10236276001) for 10 minutes and fixed with 4% paraformaldehyde for 10 minutes. Coverslips were mounted with EverBrite mounting medium (biotinum, 23001). Samples were imaged on a DeltaVision OMX V3 Blaze system (GE Healthcare) with a 60×/1.42–numerical aperture PlanApo oil immersion objective (Olympus) and pco.edge 5.5 sCMOS cameras. Image stacks were acquired over the whole cell volume, with 15 raw images per plane (five phases and three angles) for 3D structured illumination microscopy (3D-SIM)

#### Droplet Digital PCR

Reaction mixtures containing primers, template, and QX200 ddPCR EvaGreen Supermix (Bio-Rad, 186–4034) were prepared and subjected to quantification using the QX200 Droplet Digital PCR (ddPCR) System (Bio-Rad). Droplet generation and transfer of emulsified samples to PCR plates were carried out according to the manufacturer’s instructions (QX200 Droplet Generator Instruction Manual, Bio-Rad).

### Quantification and Statistical Analysis

#### Micro-Capture-C analysis

Data were analysed using the MCCuAnalysis Snakemake pipeline. Data analysis initially followed previously described methods.^87^ Adapter sequences were removed using Trim Galore (Babraham Institiute, v0.3.1), enabling the reconstruction of full-length sequences for >90% of reads. It is possible because sequencing used 300 bp reads (150 bp paired end) on material that had been sonicated to ~200 bp (FLASH (v1.2.11)^89^). Reads were first aligned using the non-stringent aligner BLAT(v35)^90^ to the tiled capture region +800 bp. The MCCuSplitter.pl script then divided the reads in the FASTQ file into sub-reads depending on the mapping by BLAT to the capture region and identified the position of sub-reads within the original reconstructed read. Bowtie 2 (v2.3.5)^91^ was used to map sub-reads were mapped to the mm10 genome build. The aligned sub-reads were analysed with MCCuAnalyser.pl, which stringently removes PCR duplicates and identifies the precise position of both short and long-range ligation junctions between sub-reads as well as the orientation of the reads relative to the junction. Subsequently data were visualised with a suite of tools to generate both conventional heatmaps and extended heatmaps for raw contact frequencies with different pixel sizes as well as matrices with contact sequence reconstruction. Multiple options for data normalisation are available in the pipeline including ICE normalisation, single cis-normalisation and tiled cis-normalisation. The full pipeline is available through the Oxford University Innovation Software store (https://process.innovation.ox.ac.uk/software/).

#### MCC peak comparison and enrichment analysis

MCC merged bigwig tracks for each viewpoint were peak called using Lanceotron.^92^ Peaks were filtered using a custom python script based on peak size, width and distance from viewpoint. Unique junctions were counted for each peak in the control and treatment conditions and were normalized using cis-unique ligation junctions for the corresponding viewpoints as previously reported.^7^ Comparisons between conditions were performed using a Student’s t-Test with multiple test correction applied using the FDR method. Peak skew was visualised using a custom script R script. MCC peaks were intersected with a variety of ChIP-seq datasets. The enrichment of ChIP-seq datasets in the statistically significantly skewed MCC peaks was calculated by the percentage of intersected significant peaks compared to percentage of intersected non-significant peaks. The log fold of the enrichment ratio was plotted using a custom script.

#### Statistical analysis of MCCu data

Comparison of MCCu data was undertaken using a bespoke statistical package, basepairC (https://github.com/jamesdalg/basepairC) specifically developed for data of this resolution. basepairC includes removal of low count and extreme outliers; library size and total count normalisation; MNase normalisation^93^; 2D segmentation with jointseg^94^; LASSO^95^ to remove to remove low count blocks and a tensor product smooth mitigate high count segments. Zero inflated negative binomial generalised linear models were used for each block to test for significant differences in ligation junction counts using an identity link.^96^ basepairC provides robust calling of biologically significant differences with base-pair resolution data because it combines stringent outlier removal and data segmentation to identify and draw inference from biologically important blocks of contacts.

#### Coarse-grained molecular dynamics simulations

The chemically specific chromatin coarse-grained model was used to investigate the biophysical properties of chromatin.^44^ This model represents every amino acid in the histone proteins explicitly represented by a bead centre on its alpha-carbon. Beads corresponding to lysine, arginine, aspartic acid, glutamic acid and histidine carry the total charge of their atomistic counterpart at pH~7. All amino acid beads have a relative hydrophobicities and diameters defined from atomistic simulations and experimentally measured data.^44^ To model acetylated lysine, we changed the charge of lysine to zero and set its molecular diameter to 6.8 Å. The histone protein core is treated with an elastic network model that enforces the secondary structure of histones in the 1KX5 crystal structure.^97^ Histone tails stemming out of the nucleosome core are treated as fully flexible polymers with bonds among consecutive residues maintained with a stiff harmonic potential and no energetic penalty for bending or torsion.

The DNA is described at a resolution of one ellipsoid per base-pair and one point charge per phosphate group (Figure 6A).^44^ The sequence-dependent mechanical properties of the DNA are defined by the Rigid Base Pair model,^98^ which approximates inter-base pair step deformations with a 6-dimensional harmonic potential in DNA helical parameter space (shift, slide, rise, roll, tilt and twist) with parameters derived from large-scale atomistic simulations of free DNA stands.^99^

Screened electrostatic interactions among all pairs of non-bonded beads are approximated using the Debye-Hückel model.^100^ Non-ionic associations among all pairs of non-bonded beads are modelled with a Leonard–Jones potential,^101^ with parameters for amino acid pairs from the Kim–Hummer model^102^ and amino acid–DNA parameters defined from atomistic simulations of nucleosomes.^103^ The model explicitly accounts for the dynamic wrapping/unwrapping of nucleosomal DNA around the histone core, and hence, nucleosome breathing, sliding, and eviction can be probed. Nucleosome wrapping in the model is determined by the energetic balance between the attractive interactions of histone residues with DNA, which decrease the energy of the nucleosome and stabilize DNA wrapping, and the mechanical deformation energy cost of bending and twisting the DNA around the histone core. By incorporating these competing forces, the model accurately reflects the plasticity of nucleosomes in response to changes in the nucleosome composition or the chromatin environment. All model parameters and energy functions can be found in Farr et al.^44^

We started the simulations in this work from an initial chromatin model that includes 16 nucleosomes with a regular nucleosome repeat length of 190 bp of DNA. Nucleosome breathing and sliding were permitted in all our simulations. To achieve adequate sampling of chromatin configurations, we used the Debye-length Hamiltonian Replica Exchange Molecular Dynamics (D-HREMD) method, which attempts exchanges between replicas with different Debye lengths using the Metropolis Criterion.^44^ We used 16 replicas with Debye lengths varying between 8 to 15 Å (equivalent to salt concentrations from 150 to 10 mM respectively). This number of replicas and the spacing among them was chosen to achieve acceptance probabilities of ~30% between consecutive replicas. Simulations were performed in the canonical ensemble (NVT) by means of the Langevin thermostat (300 K and a damping time of 10,0000 fs) and the velocity Verlet integrator in LAMMPS.^104^ We used a timestep of 40 femtoseconds, an average replica exchange frequency of 10,000 timesteps, and ran each replica for 20-30 million timesteps (880 nanoseconds). Thus, we achieved an accumulated sampling of 42 microseconds per set of 16 HREMD replicas. We recorded coordinates snapshots for each trajectory every 100,000 time-steps. For statistical analyses, we demultiplexed the trajectories and used the last 30% of steps of the replica with a Debye length of 8 Å. See Table S2 for details of the specific systems simulated.

#### Analysis of super-resolution microscopy

Raw 3D-SIM images were reconstructed with SoftWoRx 6.5.2 (GE Healthcare). Analysis of super-resolution images started with filtering of small structures using a bandpass filter. A multi-class Otsu threshold algorithm was used to classify chromatin into 7 classes.^52^ Images of histone mark foci were first thresholded based on the modulation contrast (SIMCheck^83^); The masks were created using threshold values of 12 and 14 for H3K27me3 and H3K27ac channels respectively. For all images, the channels were aligned using 3-colour EdU labelling as a reference (Chromagnon V0.92^105^). The maxima of histone mark foci were identified and mapped to their underlying chromatin class (FIJI, CLIJ^84^, MorphoLibJ^85^ and SCF-MPI libraries). Foci counts per class were normalised to class volumye.

## Supplementary Material

Supplemental information can be found online at https://doi.org/10.1016/j.cell.2025.10.013.

Supplementary Material

## Data Availability

The code for analysis of MCC data is available for academic use through the Oxford University Innovation software store (https://process.innovation.ox.ac.uk/software/p/16529a/micro-capture-c-academic/1). All other code is available through GitHub (https://github.com/jamesdalg/basepairC). Processed MCCu datasets are available on Mendeley (https://doi.org/10.17632/hvwy3kps3k). Sequencing data have been submitted to the NCBI Gene Expression Omnibus (GEO: GSE277286, GSE281416, GSE281414, GSE307191, GSE307228). Previously published data are available under the following accession codes: GEO: GSE144336, GEO: GSE153256, GEO: GSE67959,^76^ GEO: GSE97871,^77^ GEO: GSE27921,^78^ GEO: GSE30203,^79^ GEO: GSE51334,^79^ GEO: GSE44286.^80^ DNase I hypersensitivity data for erythroid^81^ and ES cells UW ENCODE^82^; GEO: GSE44286.^80^ Motif analysis was supported by ChIP-seq from GEO: GSE122589 (NANOG and SOX2), GEO: GSE181104 (GATA6), ERR263545 (OCT4), GEO: GSE98063 (POLII), GEO: GSE186349 (MED1), and GEO: GSE90895 (KLF4).

## In brief

Li et al. apply base-pair resolution Micro Capture-C ultra to map chromatin contacts between individual motifs within *cis*-regulatory elements and reveal a unified model of biophysically mediated enhancer-promoter communication.

## Highlights

- Base-pair resolution MCCu reveals complex structures within *cis*-regulatory elements
- Mediator complex components largely contribute to the fine-scale structure of promoters
- Long-range contacts are driven by transcription factor-mediated nucleosome depletion
- Chromatin structure is likely coordinated by the biophysical properties of nucleosomes
