# Supplementary material for: Mapping chromatin structure at base-pair resolution unveils a unified model of cis-regulatory element interactions
Source: Cell. Author manuscript; Available in PMC 2026 Jan 6. (PMC7618578; doi:10.1016/j.cell.2025.10.013)
Supplement: Supplementary Material [file EMS211683-supplement-Supplementary_Material.pdf]

CellPress
OPEN ACCESS

Supplemental figures

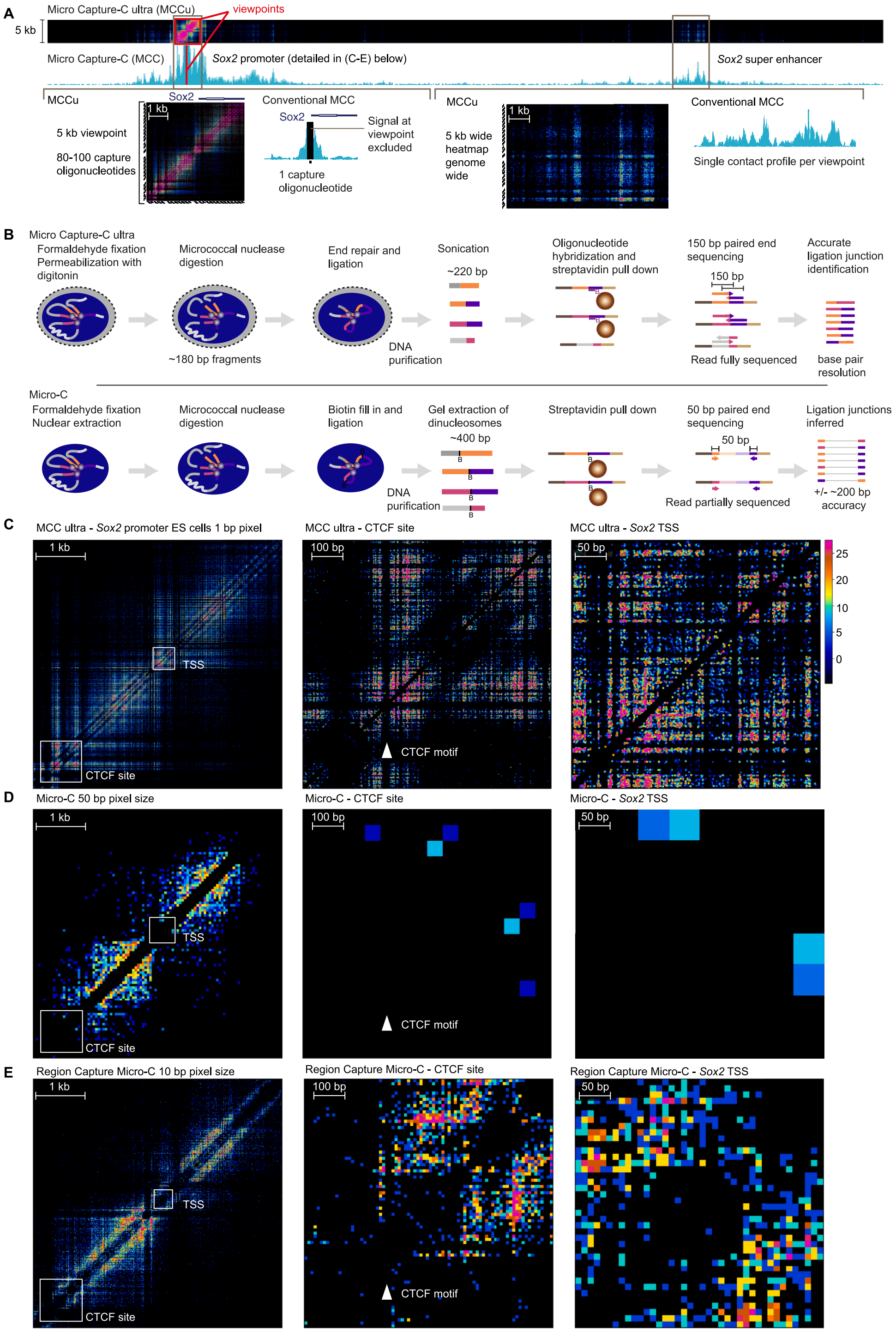

Figure S1. Comparison with other methods, related to Figure 1

(A) Comparison of MCCu experimental design and data with conventional MCC at the Sox2 locus in ES cells (region shown in Figures 1A and S1C–S1E). In MCCu, the small-scale tiling approach uses the very large amount of short-range contact data to generate heatmaps of all vs. all contacts within a 5-kb region at

(legend continued on next page)

base-pair resolution. These heatmaps can be extended outside of the captured region to allow contacts to be plotted at 5-bp resolution genome wide. In conventional MCC, a contact frequency profile is generated from a single viewpoint, and short-range contact data were excluded from analysis.

(B) Overview of MCCu in comparison with Micro-C-based methods. In MCCu, cells are fixed with 2% formaldehyde only and permeabilized with digitonin. The material is sonicated to <200 bp and sequenced with 150-bp paired-end sequencing, which allow for the whole read to be reconstructed, enabling precise identification of the ligation junction between two interacting fragments. In Micro-C, gel extraction is used to purify the dinucleosomal band, which generates a larger fragment ∼400 bp. This is then sequenced with 50-bp paired-end reads, which prevents precise identification of ligation junctions and results in limited resolution below 100 bp.

(C–E) Comparative contact maps of MCCu (C) with Micro-C (D) and Region Capture Micro-C (RCMC) (E) datasets at the 5-kb *Sox2* promoter region in mouse ES cells (raw ligation junction counts are presented as ICE normalization results in distortion of the sparse Micro-C data at this resolution). The maps include a zoom-in at an upstream 800-bp CTCF site (middle) and a 400-bp *Sox2* TSS region (right). The position of the CTCF motif is indicated with the white arrow. Note that CTCF binding prevents MNase digestion, resulting in absence of contacts directly from the motif. The contact heatmap of MCCu (C) demonstrates nanoscale interactions at 1-bp resolution. Both the Micro-C (D) and RCMC (E) datasets are displayed at their highest interpretable resolutions of 50 and 10 bp, respectively.

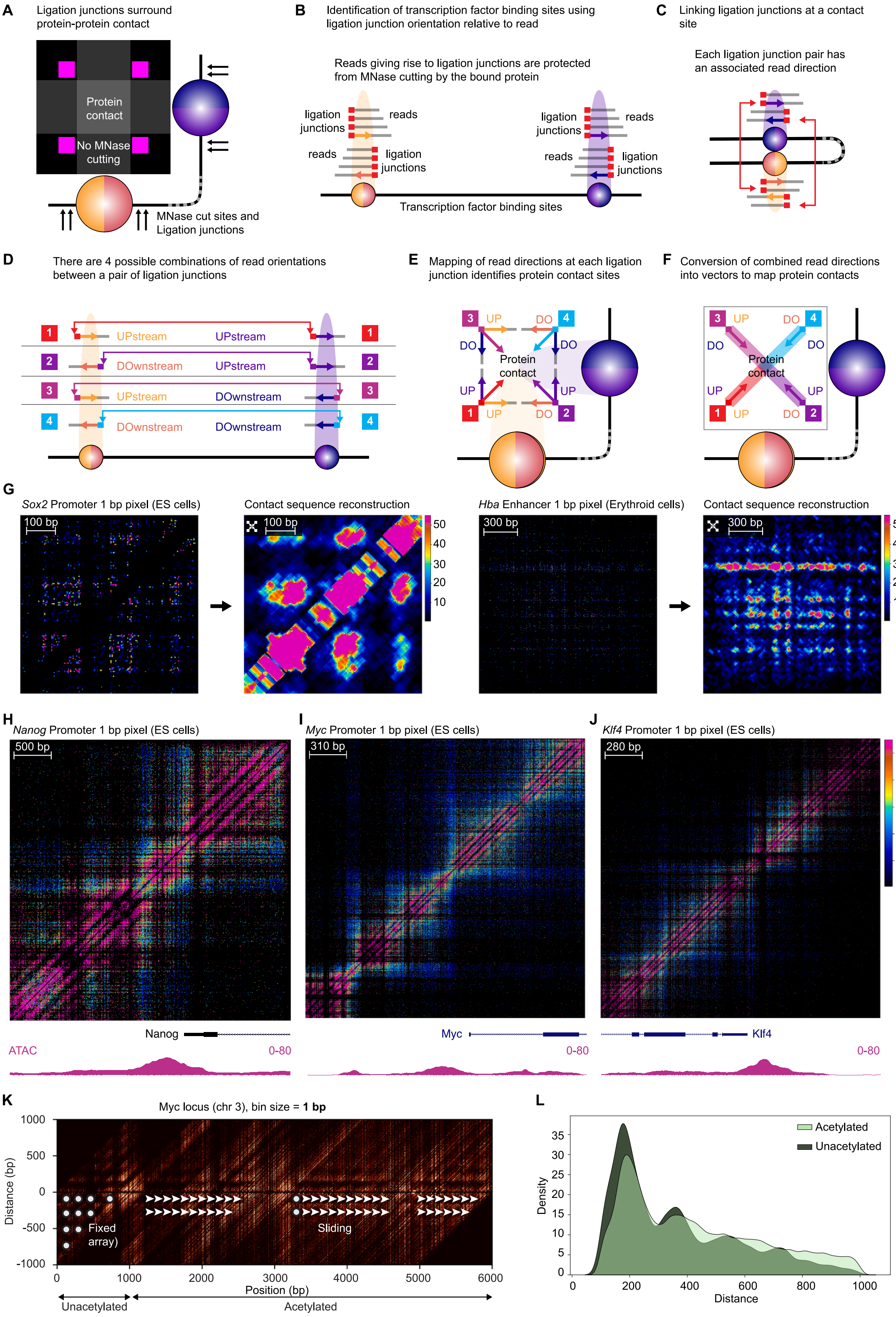

(legend on next page)

Figure S2. MCCu contact sequence reconstruction, related to Figure 2

(A–G) Overview of contact sequence reconstruction approach. (A) The DNA bound by the protein that is giving rise to the contact is protected from MNase cutting. Therefore, MNase cut sites occur adjacent to the binding sites of proteins. This results in the formation of ligation junctions around the sites of protein contact in the heatmap. (B) The direction of the protected read relative to the ligation junction can be used to identify the position of the bound proteins. (C) When two proteins are contacting each other, ligation junctions result in two pairs of genomic coordinates, and each of these has an associated read direction. (D) There are four possible combinations of read orientations for each ligation junction, depending on whether the ligation junctions are at the upstream or downstream end of the read. (E and F) Each of these combinations of orientations can be converted into a vector on the heatmap to highlight the sequences protected from MNase digestion, which are bound by proteins leading to the contact. By simultaneously mapping these orientations, the sequences bound by the proteins mediating contacts can be identified. (G) Comparison of raw data vs. contact sequence reconstruction heatmaps of *cis*-normalized directional vector density at the *Sox2* promoter and between the two key elements of the α-globin super-enhancer.

(H–J) Contact matrices for different promoter regions shows distinct partitioning patterns (ICE normalized read density). *Nanog* (left) promoter region with a single nucleosome-depleted region partitions into two domains. *Myc* promoter region (middle), featuring three nucleosome-depleted regions, partitions into multiple domains. *Klf4* promoter region (right) partitions into multiple domains.

(K) Matrix of contact frequencies by distance allows for the positioning of nucleosomes to be visualized. White circles highlight the locations of fixed nucleosomes, and white arrows indicate sliding nucleosomes with fixed internucleosome distance. Fixed nucleosomes exhibit periodic spacing of approximately 190 bp, whereas sliding nucleosomes demonstrate more flexible positioning with a sliding pattern.

(L) Metaplot comparing nucleosome spacing in acetylated and unacetylated segments from the 1,000-bp region shown in Figure S2K. The dark green line indicates that regions with fixed nucleosomes display peaks with a periodicity of ~190 bp. In contrast, the light green line shows that nucleosome spacing in the acetylated regions is associated with greater variability in ligation junction distances.

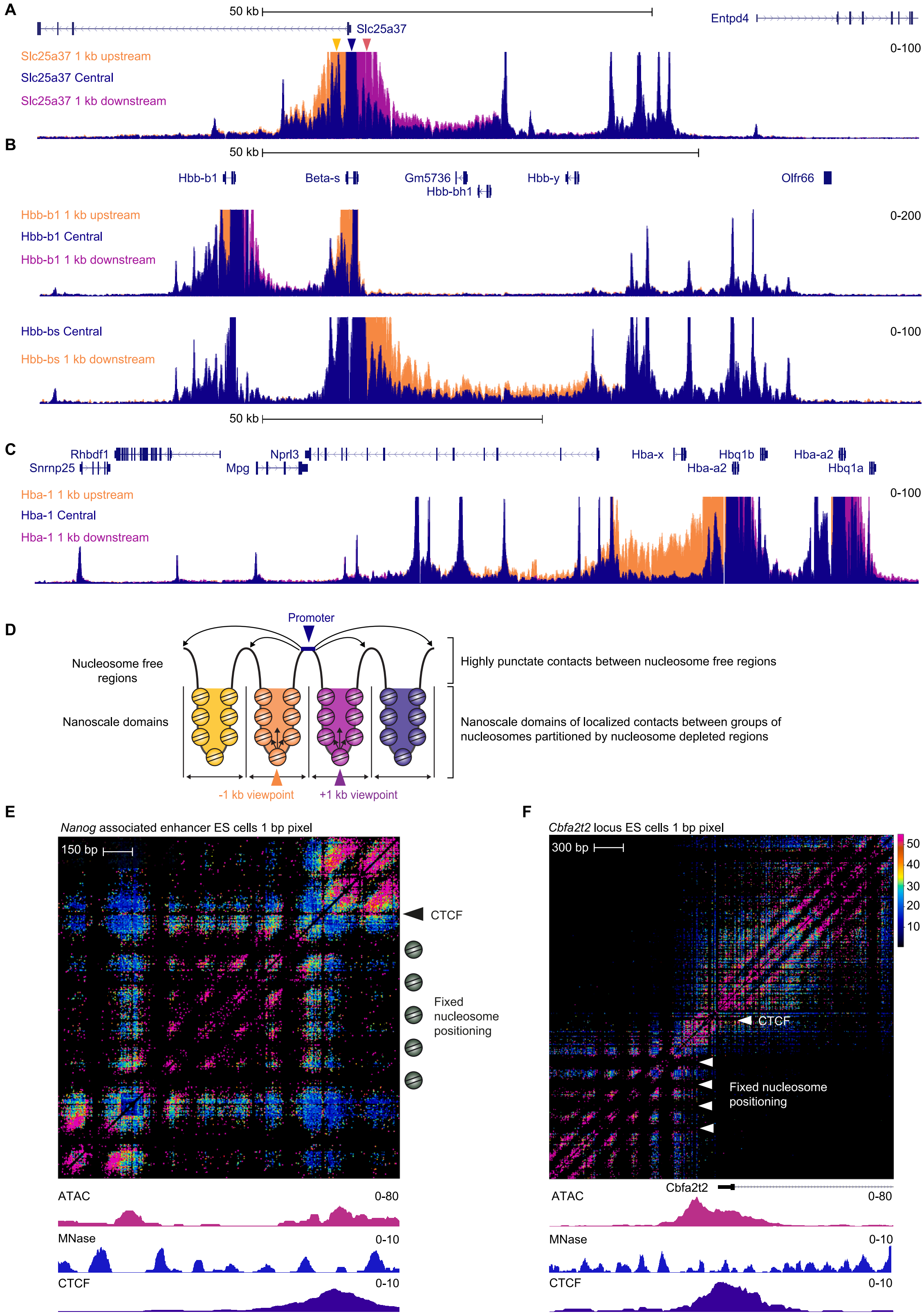

(legend on next page)

Figure S3. Interrogation of nanoscale domains and CTCF binding sites, related to Figure 3

(A–C) Conventional MCC data at the mitoferrin (*Slc25A37*), β-globin (*Hbb-b1&bs*) and α-globin (*Hba-a1&2*) loci. Probes were placed on the central nucleosome-depleted regions at the promoters (dark blue tracks) as well as 1 kb upstream (yellow tracks) and 1 kb downstream (purple tracks) (*cis*-normalized read density). The contact profiles from the nucleosome-depleted region at the center of the promoter show highly punctate contacts with other nucleosome-depleted regions at enhancers and promoters within the domain. In contrast, the off-set viewpoints show high levels of contact within the local domain, which generally extend up to the next nucleosome-depleted region.

(D) Model showing generalized contacts within individual nanoscale domains, which are insulated from one another by nucleosome-depleted regions. In contrast, highly specific contacts are observed between nucleosome-depleted regions.

(E and F) Contact matrix of CTCF sites at the *Nanog* enhancer (E) and *Cbfa2t2* promoter (F) in ES cells (1-bp pixel size, ICE normalized junction counts). ATAC-seq, MNase-seq, and CTCF ChIP-seq are shown underneath. Note the fixed spacing of nucleosomes around the sites, which manifests as fixed regions of high intensity contacts between nucleosome linker regions.

CellPress
OPEN ACCESS

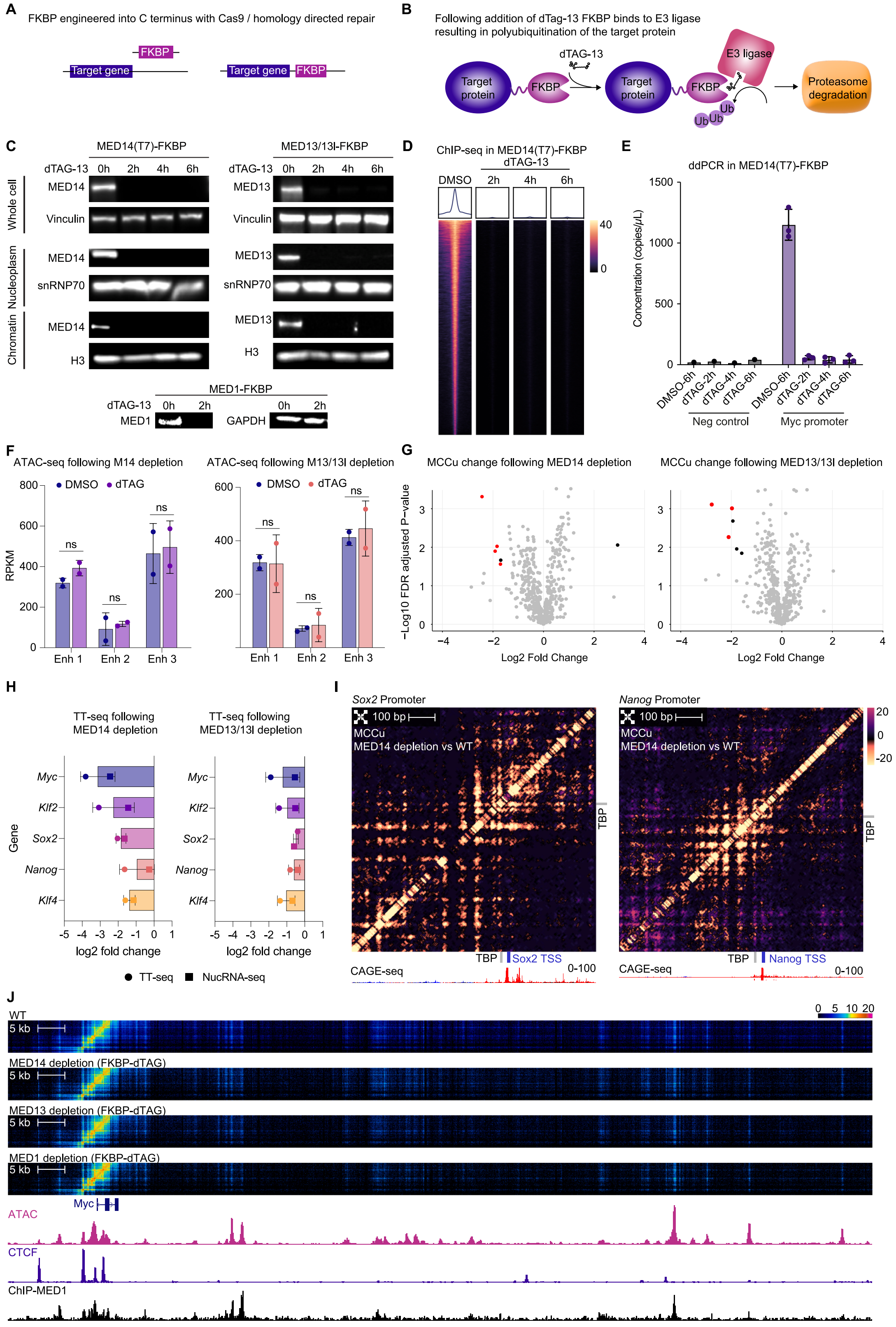

(legend on next page)

Figure S4. Degradation of Mediator subunits using the FKBP12^{F36V} dTAG degron system, related to Figure 4

(A) Schematic representation of genome engineering approach for endogenous knockin of FKBP12^{F36V} dTAG. Clones were generated by CRISPR-Cas9 and homology-directed repair (HDR). Following editing, homozygous clones were identified and expanded to generate stable FKBP12^{F36V}-expressing lines.

(B) Schematic representation of the dTAG system, demonstrating how a protein tagged with FKBP12^{F36V} can be targeted for proteasomal degradation following the introduction of the dTAG-13 small molecule.

(C) Western blot analysis of MED13 and MED14 protein levels in whole-cell lysates, nucleoplasm, and chromatin fractions following dTAG-13 treatment (500 nM) at the indicated time points. snRNP70 and histone H3 serve as loading controls for the nucleoplasmic and chromatin fractions, respectively. The western blots for MED1 show depletion in whole-cell lysates 2 h post dTAG-13 treatment (500 nM). Vinculin and GAPDH were used as loading controls.

(D) Heatmaps showing ChIP-seq for MED14 using the T7 tag in the FKBP construct at MED14 binding sites, sorted by decreasing signal, across a time course following dTAG-13 treatment (read density [CPM]).

(E) ChIP digital droplet PCR (ddPCR) analysis of MED14 occupancy at negative control region and the *Myc* promoter in MED14-FKBP mouse ES cells. DMSO-treated cells at 6 h serve as controls.

(F) Changes in chromatin accessibility measured by ATAC-seq after depletion of MED14 (left) or MED13/13l (right) in corresponding FKBP-tagged lines (data are mean ± SD, two-sided unpaired *t* test).

(G) Comparison of MCCu junction frequencies in WT and MED14-depleted (left) or MED13/13l-depleted (right) ES cells. Interactions were called in WT ES cells using Lotron MCC. Significantly skewed peaks with MED1 binding by ChIP-seq are shown in red (p value < 0.05 [adjusted for multiple testing]; absolute $\log_2$ fold change > 1.5).

(H) Bar plots showing the transcription changes measured with TT-seq and calibrated nuclear RNA-seq (cnRNA-seq) at the indicated locus in untreated (DMSO) and 2 h dTAG-13-treated MED14 and MED13/13l-FKBP cell lines.

(I) Differential contact matrices with contact sequence reconstruction within the nucleosome-depleted regions at the *Sox2* and *Nanog* promoters, in which interactions reduced in dTAG-treated cells are shown in yellow and the interactions enriched in the dTAG-treated cells are shown in purple. TATA-binding protein (TBP) (JASPAR database) and transcription start sites (TSSs) (FANTOM5[40]) are annotated.

(J) Extended heatmaps covering 160 kb at the *Myc* locus, showing the effects of dTAG-13 treatment on chromatin structure in the indicated cell line (5-bp pixel size, 6 independent replicates for each condition).

CellPress
OPEN ACCESS

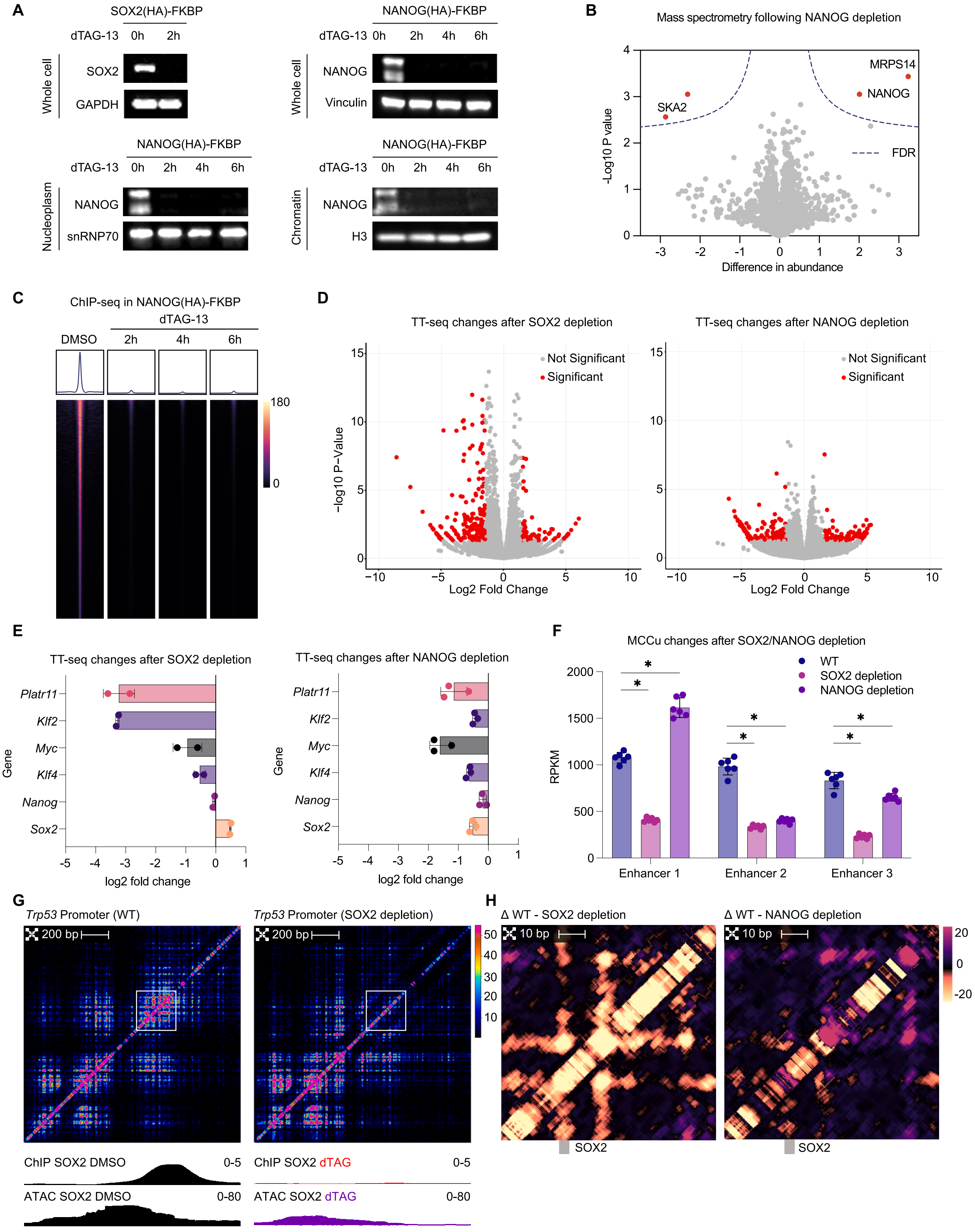

(legend on next page)

Figure S5. Degradation of transcription factors using the FKBP12^{F36V} dTAG degron system, related to Figure 5

(A) Western blot analysis demonstrating near-complete depletion of SOX2 and NANOG in whole-cell lysates upon dTAG-13 treatment (500 nM) for the indicated time points. Vinculin and GAPDH were used as loading controls. NANOG depletion was further assessed in subcellular fractions, showing loss from both the nucleoplasm and chromatin following treatment. snRNP70 and histone H3 were used as loading controls for the nucleoplasmic and chromatin fractions, respectively.

(B) Volcano plot of differentially abundant proteins identified by mass spectrometry after 2 h of dTAG-13 treatment in NANOG-FKBP mouse ES cells. Key significantly regulated proteins are highlighted in red.

(C) Heatmaps showing NANOG (hemagglutinin [HA]) ChIP-seq signal intensities at NANOG binding sites, sorted by decreasing signal, across a time course of dTAG-13 treatment (read density [CPM]).

(D) Volcano plot showing effect sizes and significance of the downregulated and upregulated (red) nascent transcribed genes measured with TT-seq after 2 h of SOX2 (left) and NANOG (right) degradation (DESeq2; p value < 0.05 and abs $\log_2$ fold change > 1.5).

(E) Bar plots showing transcriptional changes measured with TT-seq of key genes in mouse ES cells following SOX2 (left) and NANOG (right) depletion, as measured by TT-seq.

(F) Differences in ligation junction counts between the *Myc* promoter and enhancers, following depletion of SOX2 or NANOG in FKBP-tagged lines (data are mean ± SD, two-sided unpaired t test).

(G) Contact sequence reconstruction of *Trp53* locus in WT ES cells and ES SOX2-FKBP 2 h post addition of dTAG-13. ChIP-seq and ATAC-seq from DMSO and dTAG-13 treatments for SOX2-FKBP are shown below the heatmaps accordingly (*cis*-normalized directional vector density).

(H) Differential matrix with contact sequence reconstruction within the nucleosome-depleted region at a 75-bp region in *Trp53* promoter, comparing WT with SOX2 depletion or NANOG depletion (*cis*-normalized directional vector density). Degradation of SOX2 leads to a greater reduction of interactions from SOX2 motifs. Transcription factor binding sites are annotated using JASPAR.

CellPress
OPEN ACCESS

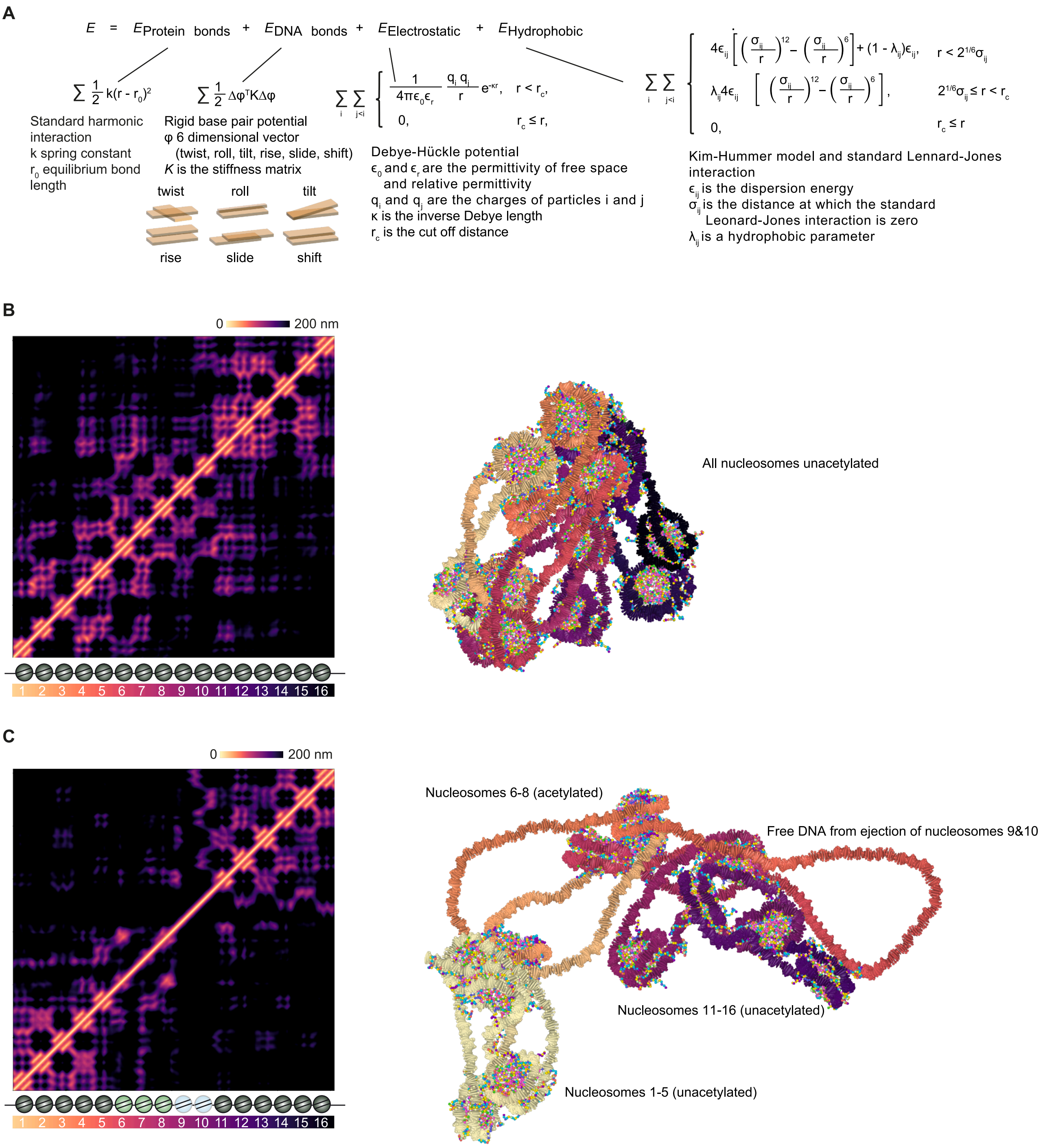

Figure S6. Molecular dynamics simulations, related to Figure 6

(A) Overview of molecular dynamics simulations. The protein bonds are modeled using a standard harmonic interaction, while a rigid base-pair potential with a 6D vector to model twist, roll, tilt, rise, slide, and shift is used to simulate bonds between DNA bases. Electrostatic interactions are modeled using the Debye-Huckle potential. The hydrophobic interactions are modeled using the Kim-Hummer model and a standard Lennard-Jones interaction.

(B) DNA distance heatmaps and representative structure for a simulation with all nucleosomes unacetylated (see Video S3).

(C) DNA distance heatmap and representative structure for a region with partial acetylation and a nucleosome-depleted region (see Video S5).

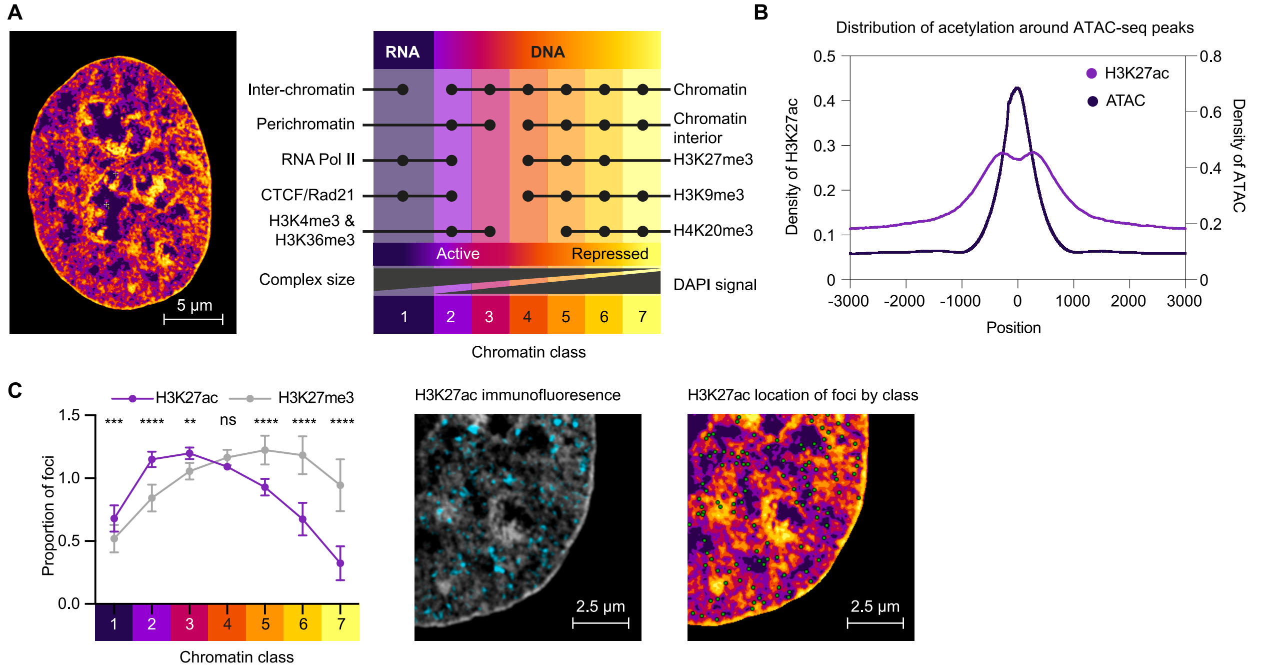

Figure S7. Integration with super-resolution microscopy, related to Figure 7

(A) Classification of chromatin using super-resolution imaging. The nucleus can be classified into one of seven classes based on the voxel intensity of the 4,6-diamidino-2-phenylindole (DAPI) signal by automated high-content image analysis. Previous studies have shown that this identifies an interchromatin compartment, which is rich in nascent RNA and polymerase; a perichromatin compartment, which contains active genes and promoters; and a central repressed core.

(B) Metaplot of the distribution of H3K27ac ChIP-seq with respect to the center of ATAC-seq peaks in mouse ES cells, showing that nucleosome-depleted regions are commonly flanked by H3K27ac.

(C) H3K27ac and H3K27me3 immunofluorescence staining of RPE1 cells imaged with 3D-structured illumination microscopy (SIM). H3K27ac is predominantly localized within the active perichromatin compartment (classes 2–4). In contrast, H3K27me3 has greater enrichment in the higher classes, which are associated with the repressed chromatin interior. The left image shows a representative single z plane of a 3D SIM image stack of immunofluorescence for H3K27ac (DAPI signal represented in gray). Note the highly localized regions of H3K27ac signal, which are juxtaposed with the interchromatin compartment, which has low DNA content and DAPI signal. The right image shows the centroid coordinates (green dots) for foci of H3K27ac superimposed on the segmented chromatin classes identified by automated high-content image analysis.
